# Enhanced Translocation and Growth of *Rhodococcus erythropolis* PR4 in the Alkane Phase of Aqueous-Alkane Two Phase Cultures Were Mediated by GroEL2 Overexpression

**DOI:** 10.1264/jsme2.ME13158

**Published:** 2014-10-11

**Authors:** Hayato Takihara, Jun Ogihara, Takao Yoshida, Shujiro Okuda, Mutsuyasu Nakajima, Noriyuki Iwabuchi, Michio Sunairi

**Affiliations:** 1 Laboratory of Molecular Microbiology, Department of Applied Biological Science, College of Bioresource Sciences, Nihon University, 1866 Kameino, Fujisawa, Kanagawa 252–8510, Japan; 2 Laboratory of Enzyme Chemistry, Department of Chemistry and Life Science, College of Bioresource Sciences, Nihon University, 1866 Kameino, Fujisawa, Kanagawa 252–8510, Japan; 3 Marine Biodiversity Research Program, Institute of Biogeosciences, Japan Agency for Marine-Earth Science and Technology (JAMSTEC), 2–15 Natsushima-cho, Yokosuka, Kanagawa 237–0061, Japan; 4 Niigata University Graduate School of Medical and Dental Sciences, 1–757 Asahimachi-dori, Chuo-ku, Niigata 951–8510, JAPAN

**Keywords:** GroEL2, organic solvent tolerance, *Rhodococcus erythropolis* PR4, alkane

## Abstract

We previously reported that *R. erythropolis* PR4 translocated from the aqueous to the alkane phase, and then grew in two phase cultures to which long-chain alkanes had been added. This was considered to be beneficial for bioremediation. In the present study, we investigated the proteins involved in the translocation of *R. erythropolis* PR4. The results of our proteogenomic analysis suggested that GroEL2 was upregulated more in cells that translocated inside of the pristane (C19) phase than in those located at the aqueous-alkane interface attached to the *n*-dodecane (C12) surface. PR4 (pK4-EL2-1) and PR4 (pK4-*Δ*EL2-1) strains were constructed to confirm the effects of the upregulation of GroEL2 in translocated cells. The expression of GroEL2 in PR4 (pK4-EL2-1) was 15.5-fold higher than that in PR4 (pK4-*Δ*EL2-1) in two phase cultures containing C12. The growth and cell surface lipophilicity of PR4 were enhanced by the introduction of pK4-EL2-1. These results suggested that the plasmid overexpression of *groEL2* in PR4 (pK4-EL2-1) led to changes in cell localization, enhanced growth, and increased cell surface lipophilicity. Thus, we concluded that the overexpression of GroEL2 may play an important role in increasing the organic solvent tolerance of *R. erythropolis* PR4 in aqueous-alkane two phase cultures.

The rhodococci, a group of Gram-positive bacteria, are useful in industrial and/or ecological applications due to their diverse range of metabolic activities. Some rhodococci can degrade organic compounds, including xenobiotics such as PCBs, while others are capable of degrading various aliphatic and aromatic hydrocarbons (3, 7). Therefore, these organisms are ideal candidates for use in the bioremediation of oil-contaminated soil.

Oil is a major environmental pollutant and consists of numerous species of hydrocarbons, such as alkanes and polyaromatics, which are small volatile and high-molecular weight hydrocarbons. Microbial activity allows the conversion of these pollutants into CO_2_ and H_2_O, which is generally considered to be an important route for the complete degradation of oil components. Bioaugmentation (spiking polluted sites with living bacteria) is a popular bioremediation method used to clean polluted soil environments. For example, high-molecular-weight hydrocarbons such as long-chain alkanes tightly adhere to soil particles in oil-contaminated soil, thereby restricting the microbial transformation of hydrocarbons due to reduced bioavailability (33). Therefore, new microbial strains capable of overcoming low bioavailability are required for effective bioremediation. A clearer understanding of the interactions between living cells and organic solvents, especially with respect to organic solvent tolerance, is needed in order to use rhodococci for these purposes.

Although a wide range of genetic and physiological studies have been reported on the effects of organic solvents on Gram-negative bacteria (27), the organic solvent tolerance mechanisms employed by Gram-positive bacteria have not yet been fully elucidated. Torres *et al.* recently summarized and reviewed some of the mechanisms employed by Gram-positive bacteria (32). Solvent-tolerance mechanisms in Gram-positive bacteria include: i) a general stress response system that acts to detoxify organic solvent molecules (25), ii) the deactivation of organic solvents by biodegradation or esterification (31), iii) changes in cell morphology and modifications to the cell surface (31), iv) cell membrane adaptations (24), and v) the efflux pump-mediated extrusion of solvent molecules (21). Mechanisms leading to organic solvent tolerance appear to be more diverse in Gram-positive bacteria than in Gram-negative bacteria. However, most studies to date have examined members of the genus *Bacillus*; information regarding the organic solvent tolerance mechanisms employed by many other Gram-positive bacteria, such as the industrially important genus *Rhodococcus*, remains limited.

In our previous studies on the interactions between *Rhodococcus* cells and organic solvents, we reported that extracellular polysaccharides (EPS) derived from *Rhodococcus* served as functional biopolymers that play major roles in regulating the interactions between the cells and organic solvents and increasing their tolerance to organic solvents (1, 10–12, 14). We also demonstrated that *R. erythropolis* PR4, an alkane-tolerant strain isolated from the Pacific Ocean (17), grew well in media containing high concentrations of pristane (13) and oil (10). We previously showed that PR4 cells completely translocated to the C19 phase in a two phase culture and subsequently multiplied, and also that close relationships existed between growth, cell localization, and alkane carbon chain length (13). The influence of the PR4 cell surface on cell localization was then determined through physicochemical characterization, which revealed that it was thermodynamically favorable for PR4 grown in hydrophobic and lipophobic C12 media to translocate from the alkane phase to the aqueous-C12 interface. In contrast, PR4 grown in hydrophobic and lipophilic C19 media preferentially entered the C19 phase, suggesting that the localization of living bacterial cells toward contaminated oils can be modulated in the bioremediation process (13).

In the present study, we attempted to clarify the molecular mechanisms underlying the translocation and alkane tolerance of PR4 utilizing a shotgun proteomic analysis, followed by molecular cloning of PR4 *groEL2*, which appeared to play a major role in these phenomena.

## Materials and Methods

### Culture conditions, bacterial growth, and cell localization and kinetic lipophilicity assays

The culture conditions and bacterial growth were as described previously (13). Briefly, 10 mL of IB medium (30) was prepared, and three different alkanes were added to the IB medium: *n*-octane (C8), *n*-dodecane (C12), and pristane (C19) at a final alkane concentration of 5% in the two phase cultures (v/v). When required, kanamycin was added to IB medium at a concentration of 200 μg mL^−1^. The two phase cultures were periodically sampled after even mixing, and viable cells in the aqueous and alkane phases were simultaneously quantified by determining the number of CFUs on IB agar plates. The cell localization and kinetic lipophilicity assays were also performed as described previously (13). Briefly, the localization of cells in a two phase culture was examined using phase-contrast microscopy (Olympus DP50, Tokyo, Japan). In the kinetic lipophilicity assay, an ionic liquid, PP13 TFSI (N-methyl-N-propylpiperidinium bis (trifluoromethanesulfonyl) imide, with a viscosity of 150 mPas at 25°C; electric conductivity, 1.5 mS cm^−1^; melting point, 12°C; water content, <50 ppm), was used to construct the two phase partitioning system.

### Genomic analyses

Most recombinant DNA techniques were performed as described by Sambrook *et al.* (26). DNA-modifying enzymes were purchased from Takara and used according to the manufacturer’s instructions. DNA restriction fragments isolated from agarose gels, as well as all PCR products, were purified using Gene Clean Kit II (Bio 101, La Jolla, CA, USA). The nucleotide sequences of plasmids, DNA fragments, and PCR products were determined in both orientations using the Dye Terminator Cycle Sequencing Kit (Perkin-Elmer, Waltham, MA, USA) and either an ABI-Prism model 377 or 3100 automatic sequencer (Perkin-Elmer). Homology between the sequences identified and those contained in DNA databases was determined using BLASTN.

### Cloning of the *groEL2* of PR4 and transformation

**Fig. 1:**
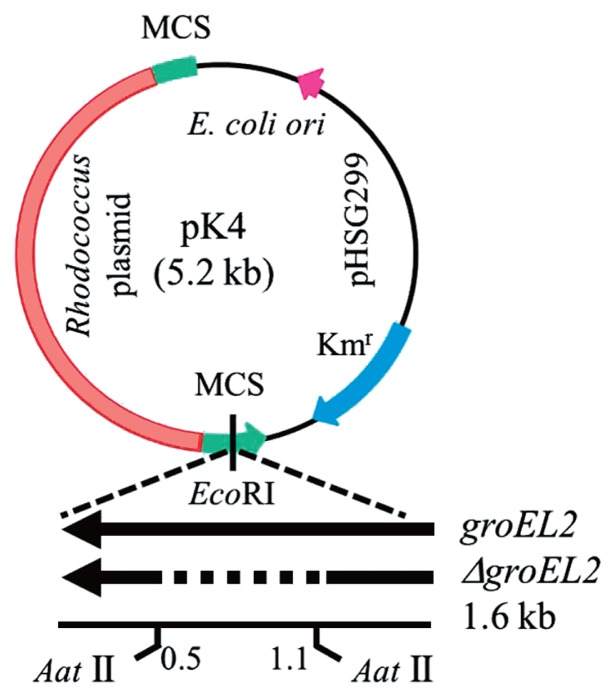
Plasmid construction scheme. The CDS of *groEL2* of *R. erythropolis* PR4 was cloned at the *Eco*RI restriction site and the resulting plasmid was modified as described in the Materials and Methods to yield the plasmids pK4-EL2-1 and pK4-*Δ*EL2-1. MCS denotes the multiple cloning sites derived from pHSG299.

A set of PCR primers (P1=ATG GCA AAG ATC ATC GCG TTC GAC; P2=TCA GAA GTC CAT GCC GCC CAT GCC GCC GGT) was constructed based on the PR4 genome and used to amplify the completed coding sequence (CDS) of *groEL2* (RER_15260), which was subsequently cloned into pCR2.1 (Invitrogen). An additional 3 PCR primers (P3=AGA AGG TCA TCC AGT CCG GCA AGC C; P4=GCT CGC CGA CAT CGC CAT CCT CAC; P5=CCA GCA GGG GCA GGT CCT TGA CCG TGG AGA TCT T) were constructed and used to determine the sequence of the cloned fragment. The resulting DNA sequence was found to be identical to that of the complete CDS of PR4 *groEL2*. The plasmid containing the cloned CDS of *groEL2* was digested using *Eco*RI, and the 1.6-kb *Eco*RI fragment was then inserted into the *Eco*RI restriction site of the *E. coli*-*Rhodococcus* shuttle vector, pK4 (8); the resulting plasmids were designated pK4-EL2-1 and pK4-EL2-2 (Fig. 1). The pK4-EL2-1 and pK4-EL2-2 plasmids contained the complete CDS of *groEL2* at the *Eco*RI restriction site downstream of the kanamycin resistance gene with the same respective forward and reverse orientations. The cloned CDS of *groEL2* was expressed by read-through from the promoter of the kanamycin resistance gene; therefore, the pK4-EL2-1 plasmid was primarily used in this study.

The intramolecular self-ligation of a DNA fragment derived from the *Aat*II digestion of pK4-EL2-1 was performed to construct a plasmid containing a defective CDS of *groEL2*. The pK4-EL2-1 plasmid was digested using *Aat*II, and the 6.3-kb fragment was subsequently extracted, purified, self-ligated, and transformed into *E. coli* HB101. The resulting pK4-*Δ*EL2-1 plasmid did not code for the hinge region of GroEL2 (Fig. 1). The pK4, pK4-EL2-1, and pK4-*Δ*EL2-1 plasmids were subsequently transformed into PR4 by electroporation (29). The *groEL2* and *ΔgroEL2* genes encoded by these plasmids were expressed by read-through from the kanamycin resistance gene promoter.

### Shotgun proteomic analysis and western blotting

Shotgun proteomic analysis and western blotting were performed as described in the supplemental materials. The shotgun proteomic analysis was independently repeated three times. Representative SDS-PAGE and western blotting are shown in Fig. S1A and S1B, respectively. The protein expression profile corresponding to Fig. S1A is shown in Table S1.

**Fig. 2:**
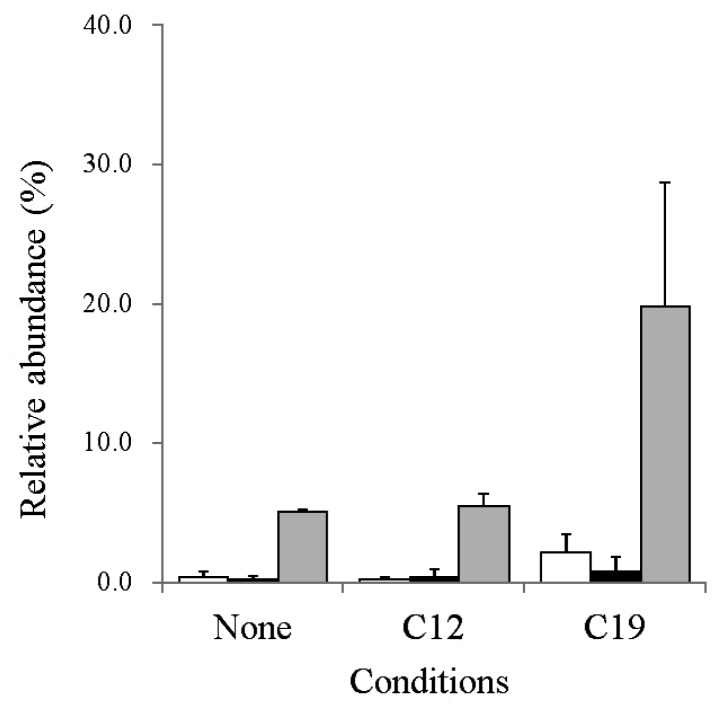
Expression of GroES, GroEL1, and GroEL2 by a shotgun proteomic analysis. “None” indicates proteins extracted from *R. erythropolis* PR4 cells grown in IB medium, “C12” indicates proteins extracted from PR4 cells grown in IB medium containing *n*-dodecane (C12), and “C19” indicates total proteins extracted from PR4 cells grown in IB medium containing pristane (C19). White, black, and light gray bars indicate GroES, GroEL1, and GroEL2, respectively. Each value is the average of 3 independent experiments. Standard deviations are presented in error bars.

The average relative abundances of GroES, GroEL1, and GroEL2 by the three shotgun proteomic analyses are summarized in Fig. 2.

## Results

### Proteogenomic analysis of *R. erythropolis* PR4

To determine differences in protein expression profiles due to cell localization between translocated and adherent PR4 cells, we first performed a shotgun proteomic analysis based on the PR4 genome. The protein expression profiles of bacteria isolated from C12 or C19-containing two phase culture media were compared.

The translational machinery and heat shock proteins appeared to be ranked higher on the protein detection list among all conditions tested (Table S1). In this experiment, we found that GroEL2 (RER_15260) accounted for 13.53% of the relative abundance of all proteins detected in PR4 cells grown in the presence of C19 (Table S1). Although GroEL2 was detected under all the conditions tested, the relative abundance in C19 appeared to be higher than in the other 2 conditions. Therefore, the average relative abundances of GroEL1 (RER_19240) and GroES (RER_19230), as well as of GroEL2, were subsequently examined. The average relative abundance of GroEL2 in the presence of C19 was 19.8 (±8.86)%, which was markedly higher than that for the other 2 conditions (Fig. 2), whereas the relative abundance of GroEL2 in the presence of C12 was similar to that in cultures grown in the absence of alkane. These results suggested that the expression of GroEL2 was upregulated in cells translocated to the C19 phase.

On the other hand, the expression of GroEL1 and GroES in cells translocated to C19 was also upregulated more than in the other 2 conditions (Fig. 2). However, the relative abundances of GroEL1 and GroES were markedly less than that of GroEL2 under the conditions tested (Fig. 2). Therefore, we focused on GroEL2 as the most abundant protein involved in cell translocation to the C19 phase in PR4.

The results of western blotting indicated that the relative expression of GroEL2 in the presence of C19 was 1.7- and 4.5-fold higher than that in cells grown in the presence of C12 and in the absence of alkane, respectively (Fig. S1B). These results supported the previous shotgun proteomic analysis results. Thus, we chose GroEL2 as the first candidate protein upregulated in cells translocated to the C19 phase.

### Cloning and expression of *groEL2* in PR4 and the effects of GroEL2 overexpression on cell localization in the presence of C12

To confirm the effects of the upregulation of GroEL2 on translocated cells in the C19 phase, we first attempted the gene disruption of *groEL2* on the PR4 chromosome, but could not obtain disruptants. We then tried the overexpression of GroEL2 in the PR4 strain. To construct transformants, the complete or defective CDS of PR4 *gorEL2* were cloned and introduced into the PR4 strain. The expression of GroEL2 from the 2 transformants, PR4 (pK4-EL2-1) and PR4 (pK4-*Δ*EL2-1), was subsequently investigated in two phase cultures to which C12 has been added. The expression of GroEL2 was examined using a shotgun proteomic approach (Table 1). The relative abundance of GroEL2 in PR4 (pK4-EL2-1) cells cultured in the presence of C12 was 18.6%, which was significantly higher than those in PR4 (pK4-*Δ*EL2-1) and PR4 (pK4) cells cultured under the same conditions, indicating that GroEL2 was overexpressed from plasmids containing the complete CDS of *groEL2* in PR4 (pK4-EL2-1).

**Fig. 3:**
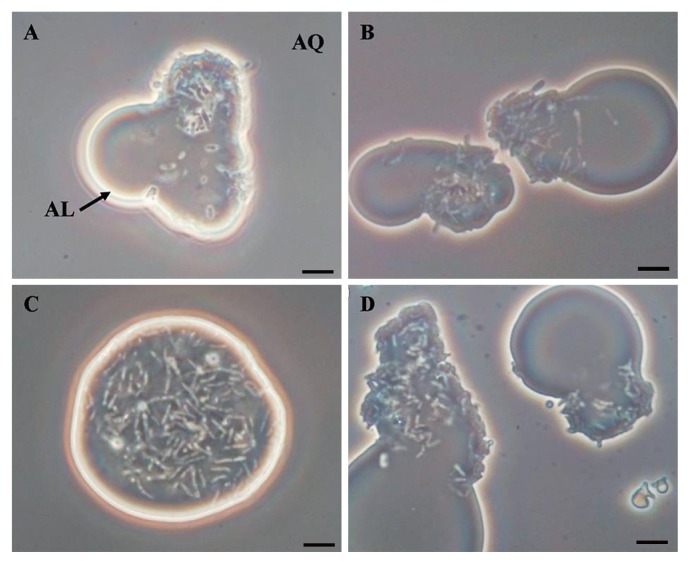
Phase-contrast micrographs illustrating the localization of *R. erythropolis* PR4 and its transformants grown in IB medium containing C12. A, PR4; B, PR4 (pK4); C, PR4 (pK4-EL2-1); D, PR4 (pK4-ΔEL2-1). “AL” and “AQ” indicate the alkane phase and aqueous supernatant phase, respectively. The culture was independently performed 3 times, 5 photographs were taken in each culture, and a typical photograph is shown here. All photographs show the same magnification. Scale bar=5 μm.

The cell localization of PR4 and its transformants was examined by phase-contrast microscopy in order to determine whether the overexpression of GroEL2 affected the cell localization of PR4 in the two phase culture in the presence of C12. Micrographs illustrating the typical localization behaviors of PR4 and its transformants cultured in the presence of C12 are shown in Fig. 3. The cells of PR4 (pK4-EL2-1) were predominately observed in the C12 phase (Fig. 3C), whereas the cells of the 3 other strains were predominately observed at the aqueous-alkane interface (Fig. 3A, 3B and 3D). These results suggested that the overexpression of GroEL2 from plasmids led to changes in the localization behavior of PR4 (pK4-EL2-1) when cultured in the presence of C12.

### Influence of alkane carbon chain length on the cell localization of transformants

**Fig. 4:**
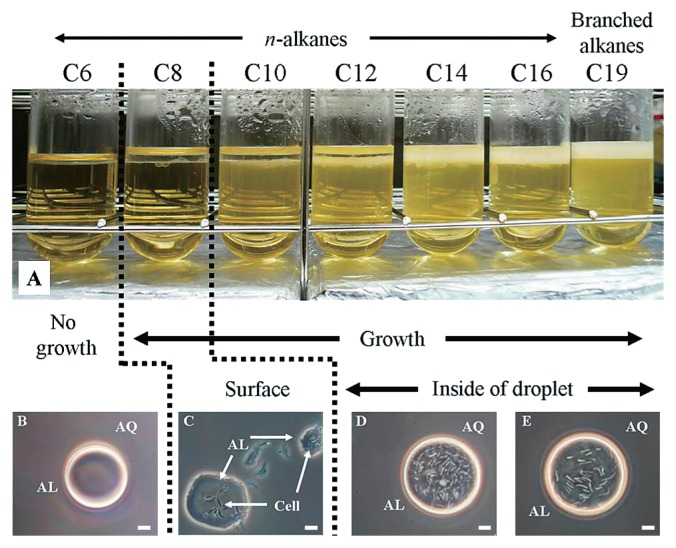
Localization of the PR4 (pK4-EL2-1) strain in two phase cultures containing alkanes of various carbon chain lengths (upper series). The lower series shows phase-contrast micrographs of hydrocarbon droplets illustrating differences in bacterial localization. “AL” and “AQ” indicate the alkane phase and aqueous supernatant phase, respectively. All photographs show the same magnification. Scale bar=5 μm.

To determine whether the overexpression of GroEL2 affected cell localization with C12 only, the effects of various alkanes on the translocation of 3 transformants, PR4 (pK4), PR4 (pK4-EL2-1), and PR4 (pK4-*Δ*EL2-1), were analyzed. The results obtained from the localization experiments of PR4 (pK4-EL2-1) in two phase cultures containing alkanes of various carbon chain lengths are shown in Fig. 4. Localization differed significantly from that of the wild type strain as described previously (13). Specifically, the alkane chain length threshold at which cells transitioned from translocation to adhesion and began to grow occurred with a shorter chain length (Fig. 4A, 4C and 4D). In the present study, PR4 (pK4-EL2-1) localized predominantly in the alkane phase in cultures containing C10 or longer alkanes and in cultures containing branched alkanes (Fig. 4D and 4E). In contrast, the cells of PR4 localized predominantly at the interface in cultures containing C10 or C12 and in the alkane phase in cultures containing long alkanes (above C14). Moreover, PR4 (pK4-EL2-1) was able to survive in the presence of C8 by adhering to the aqueous-alkane interface (Fig. 3C). On the other hand, the responses of PR4 (pK4) and PR4 (pK4-*Δ*EL2-1) to alkanes of varying chain lengths were similar to that of the wild type as described previously (13). The responses of PR4 (pK4-*Δ*EL2-1) to alkanes are shown in Fig. S2. Based on these results, we examined the effects of the overexpression of GroEL2 on the growth of the transformants in two phase cultures containing C12 in subsequent experiments.

### Effects of GroEL2 overexpression on the growth of transformants in two phase cultures containing C12

**Fig. 5:**
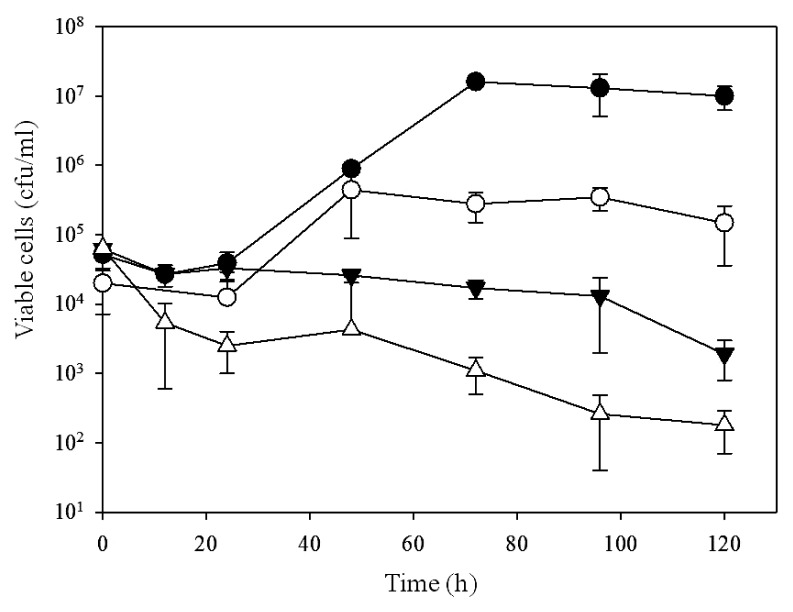
Growth of *R. erythropolis* PR4 and its transformants in two phase cultures incubated at 28°C. ●, PR4 (pK4-EL2-1) in IB medium containing C12; ○, PR4 (pK4-ΔEL2-1) in IB medium containing C12; ▼, PR4 (pK4-EL2-1) in IB medium containing C8; △, PR4 (pK4-ΔEL2-1) in IB medium containing C8. Each value is the average of at least 3 replicates from at least 3 independent experiments. Standard deviations are presented in error bars.

To investigate the relationships between GroEL2 overexpression, cell localization, and growth, PR4 (pK4-EL2-1) and PR4 (pK4-*Δ*EL2-1) were cultured in the presence of C12, and their growth was assessed at various intervals up to 120 h (Fig. 5). The initial cell densities of PR4 (pK4-EL2-1) and PR4 (pK4-*Δ*EL2-1) were 5.2×10^4^ and 2×10^4^ CFU mL^−1^, respectively. The number of viable PR4 (pK4-EL2-1) cells markedly increased after an initial lag period, ultimately reaching a maximum viable cell density of 1.6×10^7^ CFU mL^−1^. In contrast, the number of viable PR4 (pK4-*Δ*EL2-1) cells only slightly increased after an initial lag period and reached a maximum viable cell density of only 3.5×10^5^ CFU mL^−1^, even though the growth kinetics between 24 and 48 h were similar to those of PR4 (pK4-EL2-1). The introduction of the pK4 plasmid alone did not result in enhanced growth (data not shown). These results suggested that the overexpression of GroEL2 by the introduction of a plasmid harboring the complete CDS of *groEL2* led to the enhanced growth of *R. erythropolis* PR4 in the presence of the C12 alkane.

To determine whether the growth-enhancing effects of the plasmid pK4-EL2-1 were limited to the C12 alkane, we then measured the number of viable cells of the PR4 transformants in the presence of the C8 alkane, which was more toxic than C12 (Fig. 5). The PR4 (pK4-EL2-1) and PR4 (pK4-*Δ*EL2-1) transformants were cultured at initial cell densities of 6.2×10^4^ and 6.4×10^4^ CFU mL^−1^, respectively. The number of viable PR4 (pK4-EL2-1) cells remained constant (*ca.* 10^4^ CFU mL^−1^) during the first 96 h and then began to decline. In contrast, the number of viable PR4 (pK4-*Δ*EL2-1) cells decreased rapidly over the first 24 h, reached approximately 6.4×10^3^ CFU mL^−1^, and then slowly declined over the next 96 h. These results suggested that the introduction of a plasmid harboring the complete CDS of *groEL2* enhanced the survival rate of PR4 cells in the presence of C8.

### Relationship between the overexpression of *groEL2* and lipophilicity of cell surfaces

**Fig. 6:**
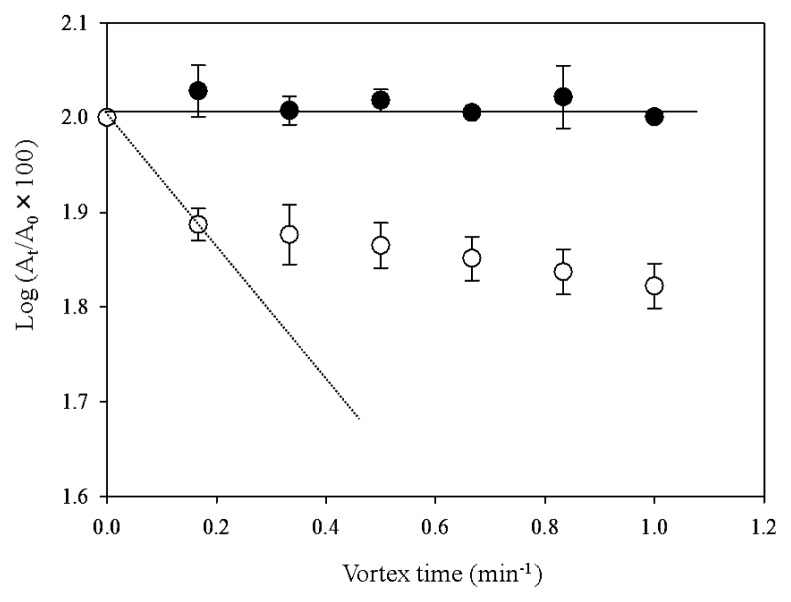
Changes in the cell density of *R. erythropolis* PR4 transformants in the *n*-hexadecane phase as a function of the vortexing time in a two phase system consisting of *n*-hexadecane and PP13 TFSI (N-methyl-N-propylpiperidinium bis (trifluoromethanesulfonyl) imide, with a viscosity of 150 mPas at 25°C; electric conductivity, 1.5 mS cm^−1^; melting point, 12°C; water content, <50 ppm). Cells were grown in IB medium containing C12 and then applied to this assay. ●, pK4-EL2-1; ○, pK4-ΔEL2-1. *A*_0_ and *A*_t_ indicate the optical densities in the alkane phase before and after mixing. Each value is the average of at least 3 replicates from at least 3 independent experiments. Standard deviations are presented in error bars. Striate and dashed lines indicate the initial removal rates (R_0_) of pK4-EL2-1 and pK4-ΔEL2-1, respectively.

A kinetic lipophilicity assay using the microbial transfer to ionic liquid (MTIL) method (13) was performed to establish how cell surface properties affected the localization behavior and growth of both transformants. The removal of each transformant from *n*-hexadecane as a function of the vortexing time when grown in the presence of C12 is shown in Fig. 6. The MTIL yields of PR4 (pK4-EL2-1) cells were significantly lower than those of PR4 (pK4-*Δ*EL2-1) cells, indicating that almost none of the PR4 (pK4-EL2-1) cells translocated from the *n*-hexadecane phase to the IL phase. In addition, the initial removal rate (R_0_) from the *n*-hexadecane phase of PR4 (pK4-EL2-1) cells grown in the presence of C12 was 0.0 min^−1^, which indicated that virtually none of the PR4 (pK4-EL2-1) cells had been removed from the *n*-hexadecane phase. In contrast, the R_0_ values for pK4-*Δ*EL2-1 and PR4 (pK4) were 0.5±0.3 min^−1^ (Fig. 6) and 0.4±0.1 min^−1^ (data not shown), respectively. These values were markedly higher than that of PR4 (pK4-EL2-1), and indicated that approximately 25% of the cells of each transformant were removed from the *n*-hexadecane phase to the ionic solution phase by the first cycle of vortexing. These results suggested that PR4 (pK4-EL2-1) cells are more lipophilic than the other transformants, and were consistent with the findings of our previous study, which showed that cells that translocated to the alkane phase were more lipophilic than cells located at the aqueous-alkane interface (13). Based on these results, we concluded that the introduction of the complete CDS of *groEL2* and subsequent overexpression of GroEL2 led to an increase in the lipophilicity of the cell surface in the presence of C12.

## Discussion

We herein demonstrated that the expression of GroEL2 was upregulated in cells that translocated to the C19 phase. The overexpression of GroEL2 from plasmids in PR4 (pK4-EL2-1) promoted an increase in the lipophilicity of the cell surface, resulting in the translocation of cells to the C12 alkane phase and enhanced growth in the presence of C12. In addition, PR4 cells were able to survive in the presence of C8 by the introduction of pK4-EL2-1. Therefore, we concluded that the overexpression of GroEL2 significantly contributed to an increase in the alkane tolerance of *R. erythropolis* PR4 in aqueous-alkane two phase cultures.

### GroEL2 overexpressed as a primary stress-response protein

In this study, we focused on GroEL2 as the most abundant protein involved in cell translocation to the C19 phase in PR4. Gram-positive bacteria have been shown to harbor multiple GroEL paralogs in their genomes, primarily GroEL1 and GroEL2 (18). Lund *et al.* (20) reported that approximately 30% of bacterial genomes contained 2 or more chaperonin genes. Of these, GroEL2 was considered to be essential and presumed to function as a housekeeping chaperone. In contrast, GroEL1 was considered to be nonessential because it was specifically expressed as the primary stress-response protein (18, 19, 23).

In the present study we found that the relative abundance of GroEL2 in translocated cells in C19 was markedly higher than that of GroEL1. On the other hand, our previous thermodynamic analyses indicated that once PR4 entered the C19 phase, as may occur due to shaking during culturing, it was thermodynamically unfavorable for cells to return to the aqueous phase because it would be associated with a positive free energy change (13). However, this result indicated that translocated cells were always surrounded by alkanes and were exposed more to alkane-induced stress during cultivation than adherent cells. The present results and our previous findings collectively indicate that a more effective stress-response system is required for translocated cells than for adherent cells. Therefore, the upregulation of GroEL2 as an overall cellular response, and not a specific cellular response, would explain why translocated cells remained in the alkane phase during cultivation. In contrast, the exposure frequency (the portion of a cell exposed to alkanes) of adherent cells was lower than that of translocated cells. The expression of GroEL2 in adherent cells was significantly lower than that of translocated cells (Fig. 2 and Table S1). This result implied that a relationship may exist between the expression of GroEL2 and exposure frequency to alkanes. Therefore, exposure frequency may be an important trigger for the upregulation of GroEL2 in PR4.

### Speculated GroEL2 function in translocated cells

The upregulation of various bacterial heat shock proteins as well as GroEL has been reported in Gram-negative bacteria in response to various stresses, such as oxidation, changes in pH, heat shock, and exposure to gamma radiation and magnetic fields (5, 6, 16). In addition, recent proteomic and transcriptomic analyses on Gram-negative bacteria showed that proteins or genes from the category of ‘heat stress response’ (*groES*, *groEL*, *grpE*, *dnaK*) were upregulated in the presence of solvents such as ethanol, butanol, toluene, and xylenes (27). These findings suggest that the upregulation of chaperones and related proteins following exposure to various stressors are widespread in Gram-negative bacteria harboring a set of GroES-EL operons. Therefore, upregulated proteins may be related to protein refolding because the presence of organic solvents in the cytoplasm and periplasm has been shown to alter protein folding in Gram-negative bacteria (27). However, it currently remains unclear how proteins of the heat stress response influence Gram-positive bacteria, which harbor multiple GroEL paralogs. Since the GroEL-ES complex refolds proteins with 14 subunits, and the molar ratio of GroEL:GroES is 1:0.5 in *E. coli* (4, 22), we deduced the molar ratios of GroEL2 and GroES in the present study by utilizing their relative abundances. The estimated molar ratio of GroEL2 and GroES in cells translocated to the C19 phase was 1:0.6 (Fig. 2), which was similar to that for *E. coli*, suggesting that the upregulated expression of GroEL2 in cells translocated to the C19 phase may be related to protein refolding.

### Effects of GroEL2 overexpression on the growth, survival, or cell surface lipophilicity of PR4 in two phase cultures

In the present study, the growth and survival of PR4 were enhanced by the overexpression of GroEL2. These results suggested that the overexpression of GroEL2 may play an important role in increasing alkane tolerance. On the other hand, we previously reported that a close relationship existed between cell localization and the growth of PR4, and also showed that PR4 grew better with translocation than with interfacial localization (13). The growth of the PR4 transformants examined in this study using C12 supports our previous conclusion.

We previously demonstrated that cell surface lipophilicity was an important factor in controlling cell localization in order to increase in alkane tolerance in aqueous-alkane two phase cultures, and also suggested that the return of previously translocated cells in C19 to the interface of aqueous-C19 was unfavorable (13). In this study, the overexpression of GroEL2 contributed to a significant increase in the cell surface lipophilicity of the translocated cells of PR4 (pK4-EL2-1), suggesting that this increase contributes to the maintenance of cell translocation in alkanes. Since the overexpression of GroEL2 in translocated cells in C12 may be related to protein refolding, GroEL2 may contribute to the maintenance of cell translocation, survival, and subsequent growth in the alkane via protein refolding in a direct and/or indirect manner.

## Conclusions

The results presented here suggest that the overexpression of GroEL2 may have greatly enhanced the alkane tolerance of *R. erythropolis* PR4 cells in the alkane phase in two phase cultures. Although further studies are needed, our results will benefit efforts to utilize living bacteria in bioremediation processes.

## Supplemental materials



## Figures and Tables

**Table 1:** Localization and GroEL2 expression in *R. erythropolis* PR4 and its transformants grown in two phase cultures containing C12

Strain	Cell localization	Relative GroEL2 expression ratio (%)
PR4	surface	4.0* (± 1.5)
PR4 (pK4)	surface	0.5* (± 0.6)
PR4 (pK4-EL2-1)	inside	18.6 (± 3.8)
PR4 (pK4-ΔEL2-1)	surface	1.2* (± 2.1)

Localization was observed using phase-contrast microscopy. The relative GroEL2 expression ratio was determined from LC-MS/MS results. Data represent the average and SD determined from 3 independent experiments. The symbol (*) represents significantly lower GroEL2 expression levels versus PR4 (pK4-EL2-1); *P*<0.05, the Student’s *t*-test).
