# Supplementary material for: Enhanced Translocation and Growth of Rhodococcus erythropolis PR4 in the Alkane Phase of Aqueous-Alkane Two Phase Cultures Were Mediated by GroEL2 Overexpression
Source: Microbes Environ. 2014 Oct 11;29(4):346–52. doi: 10.1264/jsme2.ME13158 (PMC4262357; doi:10.1264/jsme2.ME13158)
Supplement: Supplementary file 1 [file 29_346_s1.pdf]

Supplemental materials

Enhanced Translocation and Growth of *Rhodococcus erythropolis* PR4 in the Alkane Phase of Aqueous-Alkane Two Phase Cultures Were Mediated by GroEL2 Overexpression.

Hayato Takihara[1], Jun Ogihara[2], Takao Yoshida[3], Shujiro Okuda[4], Mutsuyasu Nakajima[1], Noriyuki Iwabuchi[1*], Michio Sunairi[1].

[1]Laboratory of Molecular Microbiology, Department of Applied Biological Science, College of Bioresource Sciences, Nihon University, 1866 Kameino, Fujisawa, Kanagawa 252-8510, Japan; [2]Laboratory of Enzyme Chemistry, Department of Chemistry and Life Science, College of Bioresource Sciences, Nihon University, 1866 Kameino, Fujisawa, Kanagawa 252-8510, Japan; [3]Marine Biodiversity Research Program, Institute of Biogeosciences, Japan Agency for Marine-Earth Science and Technology (JAMSTEC), 2-15 Natsushima-cho, Yokosuka, Kanagawa 237-0061, Japan; and [4]Niigata University Graduate School of Medical and Dental Sciences, 1-757 Asahimachi-dori, Chuo-ku, Niigata 951-8510, JAPAN

Supplemental materials and methods

Protein extraction and SDS-PAGE

Total cells were collected from the cultures by centrifugation or filtration using a hydrophobic filter (PTFE membrane filter, 0.5 μm, Advantec, Tokyo, Japan), and the collected cells were washed twice with MilliQ water, and resuspended in MilliQ water adjusted to final concentrations of 1×10^7 CFU mL^{-1}. The cell suspensions were centrifuged and resuspended in 100 μL of SDS-PAGE sample buffer (62.5 mM Tris, 4% SDS, 25% glycerol, 0.002% bromophenol blue, and 5% β-mercaptoethanol, pH 6.8). To obtain protein samples, the cell suspension in SDS-PAGE sample buffer were applied to sonication. When required, cell suspensions were placed in a 95°C water bath for 20 min with intermittent vortexing. The sonication and water bath steps were repeated until a liquid color becomes transparent. The resulting lysate was centrifuged at 12,000 rpm at 25°C for 15 min to pellet the cell debris. The membrane-containing supernatant was then collected and the protein concentration was determined by using a Bio-Rad Protein Assay kit (Bio-Rad, Tokyo, Japan), with bovine serum albumin as a standard. Protein isolation efficiency in each sample was checked before SDS-PAGE.

A total of 10 μg of the membrane-containing supernatant was loaded onto a 15% precast polyacrylamide gel (6.5 × 9.5 mm, Bio-Rad, Tokyo, Japan), which was run at 60 V for 4 h. Following electrophoresis, the gel was stained using an MS-compatible silver stain, as described previously (28). For downstream proteomic analysis, a single lane from the silver-stained gel was divided equally into 10 slices of 6-mm width each. Each gel slice was then cut into smaller pieces and placed into a 1.5-mL tube. The gel pieces were destained by rinsing in destaining solution (15 mM $K_3[Fe(CN)_6]$, 50 mM $Na_2S_2O_3$). Disulfide bonds were reduced by incubating samples at 56°C for 1 h in 10 mM dithiothreitol in 25 mM NH_4HCO_3. Proteins within the gel pieces were alkylated by incubation in 55 mM iodoacetamide in 25

mM NH_4HCO_3 for 45 min in the dark at room temperature. Gel pieces were then washed with 50% acetonitrile and dried in a vacuum concentrator (CVE200D, Tokyorikakikai, Tokyo, Japan). Next, 30 μL of trypsin solution (10 μg mL^{-1} in 50 mM NH_4HCO_3) prepared from modified sequencing grade trypsin (Promega, Fitchburg, WI, USA) was added to the gel pieces, and the samples were placed at 4°C for 30 min, then incubated overnight at 37°C (in-gel digestion). The resulting peptides were extracted with 50% acetonitrile containing 5% trifluoroacetic acid, and the extracted peptide mixtures were stored at -20°C until just prior to mass spectrometric analysis.

Mass spectrometry

Liquid chromatography-tandem mass spectrometry (LC-MS/MS) analysis of tryptic peptides was carried out using an LCQ Deca XP ion trap mass spectrometer (ThermoFinnigan, Waltham, MA, USA) equipped with a nano-LC electrospray ionization source (AMR, Tokyo, Japan), interfaced on-line with a capillary HPLC system (Paradigm MS4, Michrom Bioresources, Auburn, CA, USA). Prior to LC-MS/MS, samples were dried in a vacuum concentrator, redissolved in 0.1% (v/v) formic acid, and then introduced onto the analytical column using an autosampler (HTC-PAL system, CTC Analytics AG, Zwingen, Switzerland). An aliquot of peptide extract (10 μL) was first transferred onto a C8 cartridge (Peptide Captrap, Michrom Bioresources) and the peptides eluting from the cartridge were transferred onto the analytical column (L-column 2 Micro C18, 0.2 mm × 50 mm, CERI, Tokyo, Japan) by means of a switching valve. Following an initial wash with mobile phase solvent A (2% [v/v] acetonitrile, 0.1% formic acid), peptides were eluted from the analytical column using a linear gradient from 5-80% mobile phase solvent B (90% [v/v] acetonitrile, 0.1% formic acid) over a 30 min interval. The eluate was transferred directly into the electrospray ionization source of the LCQ-Deca XP ion trap mass spectrometer. Automated peak recognition,

dynamic exclusion, and daughter ion scanning of the 2 most intense ions were controlled by Xcalibur software (2, 9). Spectra were scanned over the *m/z* range 450-2,000.

Database searching and data interpretation

Database searching and data interpretation of total peptides derived from 10 slices of the silver-stained gel were performed using BioWorks 3.3 (ThermoFinnigan). All MS/MS data were analyzed using SEQUEST, a computer program that allows for correlation of experimental data with theoretical spectra generated from known protein sequences (6, 34). The criteria we used for a preliminary positive peptide identification for a doubly charged peptide were a correlation factor (Xcorr) greater than 2.5, a delta cross-correlation factor (dCn) greater than 0.1 (indicating a significant difference between the best and the next best matches reported), a high preliminary score, and a minimum of one tryptic peptide terminus. For triply charged peptides, the correlation factor threshold was set at 3.5. All peptide matches were confirmed by visual examination of the spectra, and all spectra were searched against the *R. erythropolis* PR4 genome database and/or the latest version of the NCBI public nonredundant protein database.

The % relative abundance of each identified protein by calculating the peak area of the expressed protein was obtained by the Protein Area/Height Calculation for TurboSEQUEST and MultiConsensus. Semi-quantitative information of the expressed proteins was displayed in the % total ion current (TIC) values for the peptides in the MultiConsensus Results reports. Percent TIC is the % of total ion current matched (total ion current of matched peak/total ion current of all peaks). The TIC values represent the total ion current matched for the peptide.

Data normalization

Table 1, data of shotgun proteomic analysis was normalized as follows. The conserved hypothetical protein RER_09740 was chosen as an IS for subsequent analyses because this protein was always identified under all testing conditions and in both the presence and

absence of alkane, with a relative abundance consistently greater than 1.0% in the experiments. Data regarding the relative abundance of all proteins identified in this study were adjusted based upon the abundance of RER_09740 as an IS.

Functional class assignment

KEGG Orthology (KO) including functional categories based on ortholog groups and *R. erythropolis* PR4 genome were downloaded from KEGG (15). Functional classes and the corresponding KO identifiers were extracted. Based on the KO IDs in *R. erythropolis* PR4 genome annotated by KEGG, the functional classes were assigned to each gene. The expressed proteins were categorized and listed by KO IDs.

Antibodies and western blotting

A GroEL2-specific rabbit polyclonal antibody was raised against synthetic peptides corresponding to the C-terminus (PAGDPTGGMGGMDF) of PR4 GroEL2. The peptide was conjugated to keyhole limpet hemocyanin prior to injection into rabbits. Samples containing approximately 1 μg of protein were loaded onto a 13% polyacrylamide gel for SDS-PAGE analysis. The electrophoresed proteins were transferred onto a polyvinylidine difluoride (PVDF) membrane using a semi-dry blotter (Nippon Eido, Tokyo, Japan). After blocking nonspecific protein binding using Block Ace (Dainippon Pharmaceutical, Osaka, Japan), the membrane was incubated in phosphate-buffered saline containing rabbit serum followed by alkaline phosphatase-conjugated goat anti-rabbit immunoglobulin G (Invitrogen, Carisbad, CA, USA). The bound antibodies were detected by incubating the membrane in a solution of 0.4 mg mL^{-1} nitro blue tetrazolium chloride and 0.19 mg mL^{-1} 5-bromo-4-chloro-3-indolyl phosphate in 100 mM Tris-HCl buffer (pH 9.5) containing 50 mM $MgSO_4$.

Supplemental Table

Table S1. Total protein profile.

Subclass	Pathway no. and sub-subclass	ID	Annotation	None	C12	C19
Carbohydrate metabolism	00020 Citrate cycle (TCA cycle)	RER_30680	aconitase, aconitate hydratase (acn)	0.38	0.35	2.61
	00630 Glyoxylate and dicarboxylate metabolism	RER_30680	aconitase, aconitate hydratase (acn)	0.38	0.35	2.61
	00010 Glycolysis / Gluconeogenesis	RER_30290	glyceraldehyde-3-phosphate dehydrogenase (gap)	2.22	1.42	2.25
	00020 Citrate cycle (TCA cycle)	RER_36170	dihydrolipoamide acyltransferase	1.13	0.31	1.63
	00640 Propanoate metabolism	RER_39550	methylmalonate-semialdehyde dehydrogenase (mmsA)	0.56	-	1.04
	00562 Inositol phosphate metabolism	RER_39550	methylmalonate-semialdehyde dehydrogenase (mmsA)	0.56	-	1.04
	00020 Citrate cycle (TCA cycle)	RER_20050	NADP-dependent isocitrate dehydrogenase (icd)	0.13	0.8	0.56
	00010 Glycolysis / Gluconeogenesis	RER_42830	enolase, phosphopyruvate hydratase (eno)	0.3	0.08	0.42
	00640 Propanoate metabolism	RER_45700	propionate--CoA ligase (prpE)	-	-	0.38
	00650 Butanoate metabolism	RER_23980	acetolactate synthase small subunit, acetohydroxyacid synthase (ilvN)	0.13	0.22	0.35
	00660 C5-Branched dibasic acid metabolism	RER_23980	acetolactate synthase small subunit, acetohydroxyacid synthase (ilvN)	0.13	0.22	0.35
	00030 Pentose phosphate pathway	RER_31010	6-phosphogluconate dehydrogenase (gnd)	0.1	0.09	0.33
	00020 Citrate cycle (TCA cycle)	RER_44290	succinyl-CoA synthetase beta subunit (sucC)	0.2	0.28	0.23
	00640 Propanoate metabolism	RER_44290	succinyl-CoA synthetase beta subunit (sucC)	0.2	0.28	0.23
	00660 C5-Branched dibasic acid metabolism	RER_44290	succinyl-CoA synthetase beta subunit (sucC)	0.2	0.28	0.23
	00620 Pyruvate metabolism	RER_42680	putative beta-ketoadipyl-CoA thiolase	-	0.48	0.2
	00630 Glyoxylate and dicarboxylate metabolism	RER_42680	putative beta-ketoadipyl-CoA thiolase	-	0.48	0.2
	00640 Propanoate metabolism	RER_42680	putative beta-ketoadipyl-CoA thiolase	-	0.48	0.2
	00650 Butanoate metabolism	RER_42680	putative beta-ketoadipyl-CoA thiolase	-	0.48	0.2
	00520 Amino sugar and nucleotide sugar metabolism	RER_39570	glucosamine-6-phosphate deaminase, glucosamine-6-phosphate isomerase (nagB)	-	-	0.17
	00020 Citrate cycle (TCA cycle)	RER_24250	pyruvate carboxylase (pyc)	0.04	0.22	0.17
	00620 Pyruvate metabolism	RER_24250	pyruvate carboxylase (pyc)	0.04	0.22	0.17
	00650 Butanoate metabolism	RER_56680	putative 3-hydroxybutyryl-CoA dehydrogenase (hbd)	-	0.38	0.17
	00640 Propanoate metabolism	RER_03810	fatty oxidation complex alpha subunit (fadB)	-	-	0.16

00650 Butanoate metabolism	RER_03810	fatty oxidation complex alpha subunit (fadB)	-	-	0.16
00010 Glycolysis / Gluconeogenesis	RER_15390	dihydrolipoamide dehydrogenase (lpd)	-	0.23	0.14
00020 Citrate cycle (TCA cycle)	RER_15390	dihydrolipoamide dehydrogenase (lpd)	-	0.23	0.14
00620 Pyruvate metabolism	RER_15390	dihydrolipoamide dehydrogenase (lpd)	-	0.23	0.14
00520 Amino sugar and nucleotide sugar metabolism	RER_39610	phosphotransferase system enzyme IIC component, PTS sugar-specific EIIC component	-	0.13	0.13
00010 Glycolysis / Gluconeogenesis	RER_33460	pyruvate kinase (pyk)	0.17	0.29	0.12
00620 Pyruvate metabolism	RER_33460	pyruvate kinase (pyk)	0.17	0.29	0.12
00630 Glyoxylate and dicarboxylate metabolism	RER_36230	glutamine synthetase I, glutamate--ammonia ligase (glnA, glnA1)	-	-	0.11
00620 Pyruvate metabolism	RER_32170	malate synthase (glcB, aceB)	0.05	0.37	0.11
00630 Glyoxylate and dicarboxylate metabolism	RER_32170	malate synthase (glcB, aceB)	0.05	0.37	0.11
00020 Citrate cycle (TCA cycle)	RER_20420	succinate dehydrogenase iron-sulfur protein (sdhB)	0.21	-	0.11
00650 Butanoate metabolism	RER_20420	succinate dehydrogenase iron-sulfur protein (sdhB)	0.21	-	0.11
00640 Propanoate metabolism	RER_06160	putative enoyl-CoA isomerase	-	0.02	0.11
00650 Butanoate metabolism	RER_06160	putative enoyl-CoA isomerase	-	0.02	0.11
00562 Inositol phosphate metabolism	RER_58450	myo-inositol-1-phosphate synthase (ino1)	0.2	-	0.1
00640 Propanoate metabolism	RER_39530	putative enoyl-CoA hydratase	-	-	0.09
00650 Butanoate metabolism	RER_39530	putative enoyl-CoA hydratase	-	-	0.09
00562 Inositol phosphate metabolism	RER_20190	putative 5-deoxy-glucuronate isomerase (iolB)	-	1.03	0.09
00030 Pentose phosphate pathway	RER_30440	probable transaldolase (tal)	1.24	0.37	0.08
00630 Glyoxylate and dicarboxylate metabolism	RER_21310	propionyl-CoA carboxylase beta chain, carboxyltransferase (pccB)	-	-	0.08
00640 Propanoate metabolism	RER_21310	propionyl-CoA carboxylase beta chain, carboxyltransferase (pccB)	-	-	0.08
00010 Glycolysis / Gluconeogenesis	RER_59850	branched-chain alpha-keto acid dehydrogenase E1 beta subunit	-	-	0.08
00020 Citrate cycle (TCA cycle)	RER_59850	branched-chain alpha-keto acid dehydrogenase E1 beta subunit	-	-	0.08
00620 Pyruvate metabolism	RER_59850	branched-chain alpha-keto acid dehydrogenase E1 beta subunit	-	-	0.08
00650 Butanoate metabolism	RER_59850	branched-chain alpha-keto acid dehydrogenase E1 beta subunit	-	-	0.08
00020 Citrate cycle (TCA cycle)	RER_44280	succinyl-CoA synthetase alpha subunit (sucD)	-	0.13	0.07
00640 Propanoate metabolism	RER_44280	succinyl-CoA synthetase alpha subunit (sucD)	-	0.13	0.07
00660 C5-Branched dibasic acid metabolism	RER_44280	succinyl-CoA synthetase alpha subunit (sucD)	-	0.13	0.07

00020 Citrate cycle (TCA cycle)	RER_20440	putative succinate dehydrogenase hydrophobic membrane anchor protein (sdhD)	0.05	-	0.06
00650 Butanoate metabolism	RER_20440	putative succinate dehydrogenase hydrophobic membrane anchor protein (sdhD)	0.05	-	0.06
00010 Glycolysis / Gluconeogenesis	RER_18100	aldehyde dehydrogenase	-	-	0.05
00040 Pentose and glucuronate interconversions	RER_18100	aldehyde dehydrogenase	-	-	0.05
00053 Ascorbate and aldarate metabolism	RER_18100	aldehyde dehydrogenase	-	-	0.05
00620 Pyruvate metabolism	RER_18100	aldehyde dehydrogenase	-	-	0.05
00640 Propanoate metabolism	RER_18100	aldehyde dehydrogenase	-	-	0.05
00640 Propanoate metabolism	RER_23640	putative enoyl-CoA hydratase	0.09	-	0.04
00650 Butanoate metabolism	RER_23640	putative enoyl-CoA hydratase	0.09	-	0.04
00010 Glycolysis / Gluconeogenesis	RER_28170	polyphosphate glucokinase, polyphosphate--glucose phosphotransferase (ppgK)	-	-	0.04
00520 Amino sugar and nucleotide sugar metabolism	RER_28170	polyphosphate glucokinase, polyphosphate--glucose phosphotransferase (ppgK)	-	-	0.04
00650 Butanoate metabolism	RER_23970	acetolactate synthase large subunit, acetohydroxyacid synthase (ilvB)	0.06	0.06	0.03
00660 C5-Branched dibasic acid metabolism	RER_23970	acetolactate synthase large subunit, acetohydroxyacid synthase (ilvB)	0.06	0.06	0.03
00620 Pyruvate metabolism	RER_26040	malate--quinone oxidoreductase (mqo)	-	0.24	0
00010 Glycolysis / Gluconeogenesis	RER_14120	fructose-bisphosphate aldolase class II (fba, fda)	0.37	0.1	-
00030 Pentose phosphate pathway	RER_14120	fructose-bisphosphate aldolase class II (fba, fda)	0.37	0.1	-
00051 Fructose and mannose metabolism	RER_14120	fructose-bisphosphate aldolase class II (fba, fda)	0.37	0.1	-
00620 Pyruvate metabolism	RER_04110	2-isopropylmalate synthase (leuA)	-	0.03	-
00640 Propanoate metabolism	RER_06160	putative enoyl-CoA isomerase	-	0.02	-
00650 Butanoate metabolism	RER_06160	putative enoyl-CoA isomerase	-	0.02	-
00620 Pyruvate metabolism	RER_12070	acetyl-CoA acetyltransferase, acetoacetyl-CoA thiolase	-	0.04	-
00630 Glyoxylate and dicarboxylate metabolism	RER_12070	acetyl-CoA acetyltransferase, acetoacetyl-CoA thiolase	-	0.04	-
00640 Propanoate metabolism	RER_12070	acetyl-CoA acetyltransferase, acetoacetyl-CoA thiolase	-	0.04	-
00650 Butanoate metabolism	RER_12070	acetyl-CoA acetyltransferase, acetoacetyl-CoA thiolase	-	0.04	-
00630 Glyoxylate and dicarboxylate metabolism	RER_15740	isocitrate lyase (aceA, icl)	-	0.87	-
00010 Glycolysis / Gluconeogenesis	RER_20810	putative oxidoreductase	-	0.09	-

	00020 Citrate cycle (TCA cycle)	RER_20810	putative oxidoreductase	-	0.09	-
	00620 Pyruvate metabolism	RER_20810	putative oxidoreductase	-	0.09	-
	00010 Glycolysis / Gluconeogenesis	RER_30300	phosphoglycerate kinase (pgk)	-	0.35	-
	00620 Pyruvate metabolism	RER_38940	acetyl-CoA acetyltransferase, acetoacetyl-CoA thiolase	-	0.04	-
	00630 Glyoxylate and dicarboxylate metabolism	RER_38940	acetyl-CoA acetyltransferase, acetoacetyl-CoA thiolase	-	0.04	-
	00640 Propanoate metabolism	RER_38940	acetyl-CoA acetyltransferase, acetoacetyl-CoA thiolase	-	0.04	-
	00650 Butanoate metabolism	RER_38940	acetyl-CoA acetyltransferase, acetoacetyl-CoA thiolase	-	0.04	-
	00520 Amino sugar and nucleotide sugar metabolism	RER_39610	phosphotransferase system enzyme IIC component, PTS sugar-specific EIIC component	-	0.13	-
	00010 Glycolysis / Gluconeogenesis	RER_39630	phosphotransferase system enzyme IIA component, PTS sugar-specific EIIA component	-	0.1	-
	00500 Starch and sucrose metabolism	RER_39630	phosphotransferase system enzyme IIA component, PTS sugar-specific EIIA component	-	0.1	-
	00520 Amino sugar and nucleotide sugar metabolism	RER_39630	phosphotransferase system enzyme IIA component, PTS sugar-specific EIIA component	-	0.1	-
	00650 Butanoate metabolism	RER_50070	putative 3-hydroxybutyryl-CoA dehydrogenase (hbd)	-	0.23	-
	00650 Butanoate metabolism	RER_56680	putative 3-hydroxybutyryl-CoA dehydrogenase (hbd)	-	0.38	-
	00010 Glycolysis / Gluconeogenesis	RER_10900	GTP-dependent phosphoenolpyruvate carboxykinase (pckG)	0.03	-	-
	00020 Citrate cycle (TCA cycle)	RER_10900	GTP-dependent phosphoenolpyruvate carboxykinase (pckG)	0.03	-	-
	00620 Pyruvate metabolism	RER_10900	GTP-dependent phosphoenolpyruvate carboxykinase (pckG)	0.03	-	-
	00010 Glycolysis / Gluconeogenesis	RER_30310	triose-phosphate isomerase (tpiA)	0.34	-	-
	00051 Fructose and mannose metabolism	RER_30310	triose-phosphate isomerase (tpiA)	0.34	-	-
	00562 Inositol phosphate metabolism	RER_30310	triose-phosphate isomerase (tpiA)	0.34	-	-
	00030 Pentose phosphate pathway	RER_38190	ribose-5-phosphate isomerase B, phosphoriboisomerase B (rpiB)	0.04	-	-
	00500 Starch and sucrose metabolism	RER_38890	putative glucanase (glgE)	0.06	-	-
	00020 Citrate cycle (TCA cycle)	RER_42400	fumarate hydratase class II, fumarase C (fumC)	0.09	-	-
	00030 Pentose phosphate pathway	RER_43110	phosphoribosylpyrophosphate synthase, ribose-phosphate diphosphokinase (prs)	0.27	-	-
Energy metabolism	00720 Carbon fixation pathways in prokaryotes	RER_30680	aconitase, aconitate hydratase (acn)	0.38	0.35	2.61
	00190 Oxidative phosphorylation	RER_39050	ATP synthase beta chain (atpD)	1.42	0.72	1.7
	00195 Photosynthesis	RER_39050	ATP synthase beta chain (atpD)	1.42	0.72	1.7

00190 Oxidative phosphorylation	RER_39070	ATP synthase alpha chain (atpA)	0.71	1.12	0.94
00195 Photosynthesis	RER_39070	ATP synthase alpha chain (atpA)	0.71	1.12	0.94
00920 Sulfur metabolism	RER_56710	putative NADPH--sulfite reductase flavoprotein alpha-component (cysJ)	-	-	0.64
00720 Carbon fixation pathways in prokaryotes	RER_20050	NADP-dependent isocitrate dehydrogenase (icd)	0.13	0.8	0.56
00910 Nitrogen metabolism	RER_23730	putative electron transfer flavoprotein beta subunit (etfB, fixA)	0.29	1.85	0.52
00680 Methane metabolism	RER_42830	enolase, phosphopyruvate hydratase (eno)	0.3	0.08	0.42
00190 Oxidative phosphorylation	RER_39080	ATP synthase delta chain (atpH)	0.83	0.08	0.31
00195 Photosynthesis	RER_39080	ATP synthase delta chain (atpH)	0.83	0.08	0.31
00190 Oxidative phosphorylation	RER_35970	menaquinol-cytochrome c reductase iron-sulfur protein (qcrA)	0.12	-	0.3
00190 Oxidative phosphorylation	RER_39090	ATP synthase subunit b (atpF)	-	0.1	0.27
00195 Photosynthesis	RER_39090	ATP synthase subunit b (atpF)	-	0.1	0.27
00720 Carbon fixation pathways in prokaryotes	RER_44290	succinyl-CoA synthetase beta subunit (sucC)	0.2	0.28	0.23
00920 Sulfur metabolism	RER_16530	sulfate adenylyltransferase subunit 2, ATP sulfurylase small subunit (cysD)	-	-	0.22
00910 Nitrogen metabolism	RER_23740	putative electron transfer flavoprotein alpha subunit (etfA, fixB)	0.47	0.98	0.21
00720 Carbon fixation pathways in prokaryotes	RER_42680	putative beta-ketoadipyl-CoA thiolase	-	0.48	0.2
00190 Oxidative phosphorylation	RER_31110	probable NADH dehydrogenase (ndh)	-	-	0.17
00720 Carbon fixation pathways in prokaryotes	RER_24250	pyruvate carboxylase (pyc)	0.04	0.22	0.17
00720 Carbon fixation pathways in prokaryotes	RER_03810	fatty oxidation complex alpha subunit (fadB)	-	-	0.16
00190 Oxidative phosphorylation	RER_39060	ATP synthase gamma chain (atpG)	-	-	0.14
00195 Photosynthesis	RER_39060	ATP synthase gamma chain (atpG)	-	-	0.14
00710 Carbon fixation in photosynthetic organisms	RER_33460	pyruvate kinase (pyk)	0.17	0.29	0.12
00910 Nitrogen metabolism	RER_36230	glutamine synthetase I, glutamate--ammonia ligase (glnA, glnA1)	-	-	0.11
00190 Oxidative phosphorylation	RER_20420	succinate dehydrogenase iron-sulfur protein (sdhB)	0.21	-	0.11
00720 Carbon fixation pathways in prokaryotes	RER_20420	succinate dehydrogenase iron-sulfur protein (sdhB)	0.21	-	0.11
00680 Methane metabolism	RER_24000	D-3-phosphoglycerate dehydrogenase (serA)	0.14	-	0.1
00720 Carbon fixation pathways in prokaryotes	RER_21310	propionyl-CoA carboxylase beta chain, carboxyltransferase (pccB)	-	-	0.08
00720 Carbon fixation pathways in prokaryotes	RER_44280	succinyl-CoA synthetase alpha subunit (sucD)	-	0.13	0.07
00910 Nitrogen metabolism	RER_38460	NADP-dependent glutamate dehydrogenase (gdhA)	-	-	0.07

	00190 Oxidative phosphorylation	RER_20440	putative succinate dehydrogenase hydrophobic membrane anchor protein (sdhD)	0.05	-	0.06
	00720 Carbon fixation pathways in prokaryotes	RER_20440	putative succinate dehydrogenase hydrophobic membrane anchor protein (sdhD)	0.05	-	0.06
	00710 Carbon fixation in photosynthetic organisms	RER_14120	fructose-bisphosphate aldolase class II (fba, fda)	0.37	0.1	-
	00680 Methane metabolism	RER_14120	fructose-bisphosphate aldolase class II (fba, fda)	0.37	0.1	-
	00190 Oxidative phosphorylation	RER_39040	ATP synthase epsilon chain (atpC)	0.04	0.07	-
	00195 Photosynthesis	RER_39040	ATP synthase epsilon chain (atpC)	0.04	0.07	-
	00720 Carbon fixation pathways in prokaryotes	RER_12070	acetyl-CoA acetyltransferase, acetoacetyl-CoA thiolase	-	0.04	-
	00680 Methane metabolism	RER_12250	catalase-peroxidase (katG)	-	0.08	-
	00920 Sulfur metabolism	RER_20020	homoserine O-acetyltransferase (metX, metA)	-	1.15	-
	00710 Carbon fixation in photosynthetic organisms	RER_30300	phosphoglycerate kinase (pgk)	-	0.35	-
	00720 Carbon fixation pathways in prokaryotes	RER_38940	acetyl-CoA acetyltransferase, acetoacetyl-CoA thiolase	-	0.04	-
	00920 Sulfur metabolism	RER_16520	putative sugar-phosphate phosphatase	0.22	-	-
	00710 Carbon fixation in photosynthetic organisms	RER_30310	triose-phosphate isomerase (tpiA)	0.34	-	-
	00710 Carbon fixation in photosynthetic organisms	RER_38190	ribose-5-phosphate isomerase B, phosphoriboisomerase B (rpiB)	0.04	-	-
	00720 Carbon fixation pathways in prokaryotes	RER_42400	fumarate hydratase class II, fumarase C (fumC)	0.09	-	-
Lipid metabolism	00071 Fatty acid metabolism	RER_56240	putative acetyl-CoA acyltransferase, acyl-CoA thiolase	-	-	0.57
	00592 alpha-Linolenic acid metabolism	RER_56240	putative acetyl-CoA acyltransferase, acyl-CoA thiolase	-	-	0.57
	00071 Fatty acid metabolism	RER_42680	putative beta-ketoadipyl-CoA thiolase	-	0.48	0.2
	00072 Synthesis and degradation of ketone bodies	RER_42680	putative beta-ketoadipyl-CoA thiolase	-	0.48	0.2
	00071 Fatty acid metabolism	RER_03810	fatty oxidation complex alpha subunit (fadB)	-	-	0.16
	01040 Biosynthesis of unsaturated fatty acids	RER_03810	fatty oxidation complex alpha subunit (fadB)	-	-	0.16
	00061 Fatty acid biosynthesis	RER_21210	acyl-CoA carboxylase alpha chain, biotin carboxylase/biotin carboxyl carrier protein (accA)	-	0.16	0.14
	00071 Fatty acid metabolism	RER_06160	putative enoyl-CoA isomerase	-	0.02	0.11
	00071 Fatty acid metabolism	RER_39530	putative enoyl-CoA hydratase	-	-	0.09
	00071 Fatty acid metabolism	RER_18100	aldehyde dehydrogenase	-	-	0.05
	00561 Glycerolipid metabolism	RER_18100	aldehyde dehydrogenase	-	-	0.05
	00071 Fatty acid metabolism	RER_23640	putative enoyl-CoA hydratase	0.09	-	0.04
	00071 Fatty acid metabolism	RER_06160	putative enoyl-CoA isomerase	-	0.02	-

	00071 Fatty acid metabolism	RER_12070	acetyl-CoA acetyltransferase, acetoacetyl-CoA thiolase	-	0.04	-
	00072 Synthesis and degradation of ketone bodies	RER_12070	acetyl-CoA acetyltransferase, acetoacetyl-CoA thiolase	-	0.04	-
	00071 Fatty acid metabolism	RER_38940	acetyl-CoA acetyltransferase, acetoacetyl-CoA thiolase	-	0.04	-
	00072 Synthesis and degradation of ketone bodies	RER_38940	acetyl-CoA acetyltransferase, acetoacetyl-CoA thiolase	-	0.04	-
	00071 Fatty acid metabolism	RER_40330	probable long-chain fatty-acid--CoA ligase, acyl-CoA synthetase (fadD)	-	0.21	-
	00561 Glycerolipid metabolism	RER_35800	putative acyltransferase	0.13	-	-
	00564 Glycerophospholipid metabolism	RER_35800	putative acyltransferase	0.13	-	-
Nucleotide metabolism	00230 Purine metabolism	RER_17340	DNA-directed RNA polymerase beta' chain (rpoC)	0.13	0.18	0.68
	00240 Pyrimidine metabolism	RER_17340	DNA-directed RNA polymerase beta' chain (rpoC)	0.13	0.18	0.68
	00230 Purine metabolism	RER_35310	DNA polymerase III alpha subunit (dnaE)	-	-	0.43
	00240 Pyrimidine metabolism	RER_35310	DNA polymerase III alpha subunit (dnaE)	-	-	0.43
	00230 Purine metabolism	RER_16530	sulfate adenylyltransferase subunit 2, ATP sulfurylase small subunit (cysD)	-	-	0.22
	00230 Purine metabolism	RER_19330	inosine-5'-monophosphate dehydrogenase, IMP dehydrogenase (guaB)	-	0.08	0.21
	00230 Purine metabolism	RER_17330	DNA-directed RNA polymerase beta chain (rpoB)	-	0.37	0.18
	00240 Pyrimidine metabolism	RER_17330	DNA-directed RNA polymerase beta chain (rpoB)	-	0.37	0.18
	00230 Purine metabolism	RER_26440	guanosine pentaphosphate synthetase/polynucleotide phosphorylase, polyribonucleotide nucleotidyltransferase (gpsI, pnp)	0.05	0.34	0.12
	00240 Pyrimidine metabolism	RER_26440	guanosine pentaphosphate synthetase/polynucleotide phosphorylase, polyribonucleotide nucleotidyltransferase (gpsI, pnp)	0.05	0.34	0.12
	00230 Purine metabolism	RER_33460	pyruvate kinase (pyk)	0.17	0.29	0.12
	00230 Purine metabolism	RER_18810	DNA-directed RNA polymerase alpha chain (rpoA)	0.1	-	0.09
	00240 Pyrimidine metabolism	RER_18810	DNA-directed RNA polymerase alpha chain (rpoA)	0.1	-	0.09
	00230 Purine metabolism	RER_10470	phosphoribosylformylglycinamidine synthase I, formylglycinamide ribonucleotide amidotransferase (purQ)	-	0.26	-
	00230 Purine metabolism	RER_43110	phosphoribosylpyrophosphate synthase, ribose-phosphate diphosphokinase (prs)	0.27	-	-
Amino acid metabolism	00300 Lysine biosynthesis	RER_30680	aconitase, aconitate hydratase (acn)	0.38	0.35	2.61
	00290 Valine, leucine and isoleucine biosynthesis	RER_23990	ketol-acid reductoisomerase, acetohydroxy acid	1.58	0.68	1.71

		isomeroreductase (ilvC)			
00310 Lysine degradation	RER_36170	dihydrolipoamide acyltransferase	1.13	0.31	1.63
00280 Valine, leucine and isoleucine degradation	RER_39550	methylmalonate-semialdehyde dehydrogenase (mmsA)	0.56	-	1.04
00280 Valine, leucine and isoleucine degradation	RER_56240	putative acetyl-CoA acyltransferase, acyl-CoA thiolase	-	-	0.57
00290 Valine, leucine and isoleucine biosynthesis	RER_23980	acetolactate synthase small subunit, acetohydroxyacid synthase (ilvN)	0.13	0.22	0.35
00280 Valine, leucine and isoleucine degradation	RER_42680	putative beta-ketoadipyl-CoA thiolase	-	0.48	0.2
00310 Lysine degradation	RER_42680	putative beta-ketoadipyl-CoA thiolase	-	0.48	0.2
00380 Tryptophan metabolism	RER_42680	putative beta-ketoadipyl-CoA thiolase	-	0.48	0.2
00360 Phenylalanine metabolism	RER_56680	putative 3-hydroxybutyryl-CoA dehydrogenase (hbd)	-	0.38	0.17
00280 Valine, leucine and isoleucine degradation	RER_03810	fatty oxidation complex alpha subunit (fadB)	-	-	0.16
00310 Lysine degradation	RER_03810	fatty oxidation complex alpha subunit (fadB)	-	-	0.16
00380 Tryptophan metabolism	RER_03810	fatty oxidation complex alpha subunit (fadB)	-	-	0.16
00260 Glycine, serine and threonine metabolism	RER_15390	dihydrolipoamide dehydrogenase (lpd)	-	0.23	0.14
00280 Valine, leucine and isoleucine degradation	RER_15390	dihydrolipoamide dehydrogenase (lpd)	-	0.23	0.14
00350 Tyrosine metabolism	RER_54270	putative phenol hydroxylase	-	-	0.14
00250 Alanine, aspartate and glutamate metabolism	RER_36230	glutamine synthetase I, glutamate--ammonia ligase (glnA, glnA1)	-	-	0.11
00330 Arginine and proline metabolism	RER_36230	glutamine synthetase I, glutamate--ammonia ligase (glnA, glnA1)	-	-	0.11
00280 Valine, leucine and isoleucine degradation	RER_06160	putative enoyl-CoA isomerase	-	0.02	0.11
00310 Lysine degradation	RER_06160	putative enoyl-CoA isomerase	-	0.02	0.11
00360 Phenylalanine metabolism	RER_06160	putative enoyl-CoA isomerase	-	0.02	0.11
00380 Tryptophan metabolism	RER_06160	putative enoyl-CoA isomerase	-	0.02	0.11
00260 Glycine, serine and threonine metabolism	RER_24000	D-3-phosphoglycerate dehydrogenase (serA)	0.14	-	0.1
00280 Valine, leucine and isoleucine degradation	RER_39530	putative enoyl-CoA hydratase	-	-	0.09
00310 Lysine degradation	RER_39530	putative enoyl-CoA hydratase	-	-	0.09
00360 Phenylalanine metabolism	RER_39530	putative enoyl-CoA hydratase	-	-	0.09
00380 Tryptophan metabolism	RER_39530	putative enoyl-CoA hydratase	-	-	0.09
00280 Valine, leucine and isoleucine degradation	RER_21310	propionyl-CoA carboxylase beta chain, carboxyltransferase (pccB)	-	-	0.08
00300 Lysine biosynthesis	RER_39230	diaminopimelate decarboxylase (lysA)	-	-	0.08

00250 Alanine, aspartate and glutamate metabolism	RER_38460	NADP-dependent glutamate dehydrogenase (gdhA)	-	-	0.07
00330 Arginine and proline metabolism	RER_38460	NADP-dependent glutamate dehydrogenase (gdhA)	-	-	0.07
00290 Valine, leucine and isoleucine biosynthesis	RER_24010	3-isopropylmalate dehydrogenase (leuB)	-	-	0.06
00280 Valine, leucine and isoleucine degradation	RER_18100	aldehyde dehydrogenase	-	-	0.05
00310 Lysine degradation	RER_18100	aldehyde dehydrogenase	-	-	0.05
00330 Arginine and proline metabolism	RER_18100	aldehyde dehydrogenase	-	-	0.05
00340 Histidine metabolism	RER_18100	aldehyde dehydrogenase	-	-	0.05
00380 Tryptophan metabolism	RER_18100	aldehyde dehydrogenase	-	-	0.05
00280 Valine, leucine and isoleucine degradation	RER_23640	putative enoyl-CoA hydratase	0.09	-	0.04
00310 Lysine degradation	RER_23640	putative enoyl-CoA hydratase	0.09	-	0.04
00360 Phenylalanine metabolism	RER_23640	putative enoyl-CoA hydratase	0.09	-	0.04
00380 Tryptophan metabolism	RER_23640	putative enoyl-CoA hydratase	0.09	-	0.04
00290 Valine, leucine and isoleucine biosynthesis	RER_23970	acetolactate synthase large subunit, acetohydroxyacid synthase (ilvB)	0.06	0.06	0.03
00270 Cysteine and methionine metabolism	RER_30110	S-adenosylmethionine synthetase, methionine adenosyltransferase (metK)	0.5	0.28	-
00290 Valine, leucine and isoleucine biosynthesis	RER_04110	2-isopropylmalate synthase (leuA)	-	0.03	-
00260 Glycine, serine and threonine metabolism	RER_04140	aspartokinase (ask)	-	0.04	-
00270 Cysteine and methionine metabolism	RER_04140	aspartokinase (ask)	-	0.04	-
00300 Lysine biosynthesis	RER_04140	aspartokinase (ask)	-	0.04	-
00280 Valine, leucine and isoleucine degradation	RER_06160	putative enoyl-CoA isomerase	-	0.02	-
00310 Lysine degradation	RER_06160	putative enoyl-CoA isomerase	-	0.02	-
00360 Phenylalanine metabolism	RER_06160	putative enoyl-CoA isomerase	-	0.02	-
00380 Tryptophan metabolism	RER_06160	putative enoyl-CoA isomerase	-	0.02	-
00280 Valine, leucine and isoleucine degradation	RER_12070	acetyl-CoA acetyltransferase, acetoacetyl-CoA thiolase	-	0.04	-
00310 Lysine degradation	RER_12070	acetyl-CoA acetyltransferase, acetoacetyl-CoA thiolase	-	0.04	-
00380 Tryptophan metabolism	RER_12070	acetyl-CoA acetyltransferase, acetoacetyl-CoA thiolase	-	0.04	-
00360 Phenylalanine metabolism	RER_12250	catalase-peroxidase (katG)	-	0.08	-
00380 Tryptophan metabolism	RER_12250	catalase-peroxidase (katG)	-	0.08	-
00270 Cysteine and methionine metabolism	RER_20020	homoserine O-acetyltransferase (metX, metA)	-	1.15	-
00260 Glycine, serine and threonine metabolism	RER_20810	putative oxidoreductase	-	0.09	-

	00280 Valine, leucine and isoleucine degradation	RER_20810	putative oxidoreductase	-	0.09	-
	00270 Cysteine and methionine metabolism	RER_21660	adenosylhomocysteinase, S-adenosyl-L-homocysteine hydrolase (ahcY)	-	0.46	-
	00280 Valine, leucine and isoleucine degradation	RER_38940	acetyl-CoA acetyltransferase, acetoacetyl-CoA thiolase	-	0.04	-
	00310 Lysine degradation	RER_38940	acetyl-CoA acetyltransferase, acetoacetyl-CoA thiolase	-	0.04	-
	00380 Tryptophan metabolism	RER_38940	acetyl-CoA acetyltransferase, acetoacetyl-CoA thiolase	-	0.04	-
	00270 Cysteine and methionine metabolism	RER_48100	putative thiosulfate sulfurtransferase, rhodanese	-	0.15	-
	00360 Phenylalanine metabolism	RER_50070	putative 3-hydroxybutyryl-CoA dehydrogenase (hbd)	-	0.23	-
	00360 Phenylalanine metabolism	RER_56680	putative 3-hydroxybutyryl-CoA dehydrogenase (hbd)	-	0.38	-
	00330 Arginine and proline metabolism	RER_57110	putative amidase	-	0.07	-
	00360 Phenylalanine metabolism	RER_57110	putative amidase	-	0.07	-
	00380 Tryptophan metabolism	RER_57110	putative amidase	-	0.07	-
	00340 Histidine metabolism	RER_23050	histidinol-phosphate phosphatase, histidinol-phosphatase (hisN)	0.38	-	-
	00300 Lysine biosynthesis	RER_27320	dihydrodipicolinate synthase (dapA)	0.14	-	-
	00340 Histidine metabolism	RER_34520	histidinol dehydrogenase (hisD)	0.02	-	-
	00280 Valine, leucine and isoleucine degradation	RER_39520	3-hydroxyisobutyrate dehydrogenase (mmsB)	0.17	-	-
Metabolism of other amino acids	00410 beta-Alanine metabolism	RER_39550	methylmalonate-semialdehyde dehydrogenase (mmsA)	0.56	-	1.04
	00480 Glutathione metabolism	RER_20050	NADP-dependent isocitrate dehydrogenase (icd)	0.13	0.8	0.56
	00480 Glutathione metabolism	RER_31010	6-phosphogluconate dehydrogenase (gnd)	0.1	0.09	0.33
	00450 Selenocompound metabolism	RER_16530	sulfate adenylyltransferase subunit 2, ATP sulfurylase small subunit (cysD)	-	-	0.22
	00410 beta-Alanine metabolism	RER_03810	fatty oxidation complex alpha subunit (fadB)	-	-	0.16
	00410 beta-Alanine metabolism	RER_06160	putative enoyl-CoA isomerase	-	0.02	0.11
	00410 beta-Alanine metabolism	RER_39530	putative enoyl-CoA hydratase	-	-	0.09
	00410 beta-Alanine metabolism	RER_18100	aldehyde dehydrogenase	-	-	0.05
	00410 beta-Alanine metabolism	RER_23640	putative enoyl-CoA hydratase	0.09	-	0.04
	00410 beta-Alanine metabolism	RER_06160	putative enoyl-CoA isomerase	-	0.02	-
Glycan biosynthesis and metabolism	00550 Peptidoglycan biosynthesis	RER_60150	conserved hypothetical membrane protein	0.32	-	-
	00770 Pantothenate and CoA biosynthesis	RER_23990	ketol-acid reductoisomerase, acetohydroxy acid isomeroreductase (ilvC)	1.58	0.68	1.71

Metabolism of cofactors and vitamins	00770 Pantothenate and CoA biosynthesis	RER_23980	acetolactate synthase small subunit, acetohydroxyacid synthase (ilvN)	0.13	0.22	0.35
	00750 Vitamin B6 metabolism	RER_29090	probable pyridoxal phosphate synthase component Pdx1, PLP synthase subunit (pdx1, pdxS)	-	-	0.05
	00770 Pantothenate and CoA biosynthesis	RER_23970	acetolactate synthase large subunit, acetohydroxyacid synthase (ilvB)	0.06	0.06	0.03
	00740 Riboflavin metabolism	RER_30230	6,7-dimethyl-8-ribityllumazine synthase, riboflavin synthase beta chain (ribH, ribE)	-	-	0.02
	00750 Vitamin B6 metabolism	RER_32350	phosphomethylpyrimidine kinase (thiD)	-	0.18	-
Metabolism of terpenoids and polyketides	00281 Geraniol degradation	RER_56240	putative acetyl-CoA acyltransferase, acyl-CoA thiolase	-	-	0.57
	00900 Terpenoid backbone biosynthesis	RER_42680	putative beta-ketoadipyl-CoA thiolase	-	0.48	0.2
	00903 Limonene and pinene degradation	RER_03810	fatty oxidation complex alpha subunit (fadB)	-	-	0.16
	00281 Geraniol degradation	RER_03810	fatty oxidation complex alpha subunit (fadB)	-	-	0.16
	00903 Limonene and pinene degradation	RER_06160	putative enoyl-CoA isomerase	-	0.02	0.11
	00281 Geraniol degradation	RER_06160	putative enoyl-CoA isomerase	-	0.02	0.11
	00903 Limonene and pinene degradation	RER_39530	putative enoyl-CoA hydratase	-	-	0.09
	00281 Geraniol degradation	RER_39530	putative enoyl-CoA hydratase	-	-	0.09
	00903 Limonene and pinene degradation	RER_18100	aldehyde dehydrogenase	-	-	0.05
	00903 Limonene and pinene degradation	RER_23640	putative enoyl-CoA hydratase	0.09	-	0.04
	00281 Geraniol degradation	RER_23640	putative enoyl-CoA hydratase	0.09	-	0.04
	00903 Limonene and pinene degradation	RER_06160	putative enoyl-CoA isomerase	-	0.02	-
	00281 Geraniol degradation	RER_06160	putative enoyl-CoA isomerase	-	0.02	-
	00900 Terpenoid backbone biosynthesis	RER_12070	acetyl-CoA acetyltransferase, acetoacetyl-CoA thiolase	-	0.04	-
	00900 Terpenoid backbone biosynthesis	RER_38940	acetyl-CoA acetyltransferase, acetoacetyl-CoA thiolase	-	0.04	-
Biosynthesis of other secondary metabolites	00521 Streptomycin biosynthesis	RER_58450	myo-inositol-1-phosphate synthase (ino1)	0.2	-	0.1
	00940 Phenylpropanoid biosynthesis	RER_12250	catalase-peroxidase (katG)	-	0.08	-
Xenobiotics biodegradation and metabolism	00362 Benzoate degradation	RER_56240	putative acetyl-CoA acyltransferase, acyl-CoA thiolase	-	-	0.57
	00642 Ethylbenzene degradation	RER_56240	putative acetyl-CoA acyltransferase, acyl-CoA thiolase	-	-	0.57
	00983 Drug metabolism - other enzymes	RER_19330	inosine-5'-monophosphate dehydrogenase, IMP dehydrogenase (guaB)	-	0.08	0.21
	00362 Benzoate degradation	RER_42680	putative beta-ketoadipyl-CoA thiolase	-	0.48	0.2

	00362 Benzoate degradation	RER_56680	putative 3-hydroxybutyryl-CoA dehydrogenase (hbd)	-	0.38	0.17
	00930 Caprolactam degradation	RER_03810	fatty oxidation complex alpha subunit (fadB)	-	-	0.16
	00623 Toluene degradation	RER_20420	succinate dehydrogenase iron-sulfur protein (sdhB)	0.21	-	0.11
	00362 Benzoate degradation	RER_06160	putative enoyl-CoA isomerase	-	0.02	0.11
	00627 Aminobenzoate degradation	RER_06160	putative enoyl-CoA isomerase	-	0.02	0.11
	00930 Caprolactam degradation	RER_06160	putative enoyl-CoA isomerase	-	0.02	0.11
	00362 Benzoate degradation	RER_39530	putative enoyl-CoA hydratase	-	-	0.09
	00627 Aminobenzoate degradation	RER_39530	putative enoyl-CoA hydratase	-	-	0.09
	00930 Caprolactam degradation	RER_39530	putative enoyl-CoA hydratase	-	-	0.09
	00623 Toluene degradation	RER_20440	putative succinate dehydrogenase hydrophobic membrane anchor protein (sdhD)	0.05	-	0.06
	00625 Chloroalkane and chloroalkene degradation	RER_18100	aldehyde dehydrogenase	-	-	0.05
	00362 Benzoate degradation	RER_23640	putative enoyl-CoA hydratase	0.09	-	0.04
	00627 Aminobenzoate degradation	RER_23640	putative enoyl-CoA hydratase	0.09	-	0.04
	00930 Caprolactam degradation	RER_23640	putative enoyl-CoA hydratase	0.09	-	0.04
	00362 Benzoate degradation	RER_06160	putative enoyl-CoA isomerase	-	0.02	-
	00627 Aminobenzoate degradation	RER_06160	putative enoyl-CoA isomerase	-	0.02	-
	00930 Caprolactam degradation	RER_06160	putative enoyl-CoA isomerase	-	0.02	-
	00984 Steroid degradation	RER_07540	probable 3-ketosteroid 9alpha-hydroxylase component KshA, terminal oxygenase (kshA)	-	1.57	-
	00362 Benzoate degradation	RER_12070	acetyl-CoA acetyltransferase, acetoacetyl-CoA thiolase	-	0.04	-
	00362 Benzoate degradation	RER_38940	acetyl-CoA acetyltransferase, acetoacetyl-CoA thiolase	-	0.04	-
	00362 Benzoate degradation	RER_50070	putative 3-hydroxybutyryl-CoA dehydrogenase (hbd)	-	0.23	-
	00362 Benzoate degradation	RER_56680	putative 3-hydroxybutyryl-CoA dehydrogenase (hbd)	-	0.38	-
	00627 Aminobenzoate degradation	RER_57110	putative amidase	-	0.07	-
	00643 Styrene degradation	RER_57110	putative amidase	-	0.07	-
Transcription	03020 RNA polymerase	RER_17340	DNA-directed RNA polymerase beta' chain (rpoC)	0.13	0.18	0.68
	03020 RNA polymerase	RER_17330	DNA-directed RNA polymerase beta chain (rpoB)	-	0.37	0.18
	03020 RNA polymerase	RER_18810	DNA-directed RNA polymerase alpha chain (rpoA)	0.1	-	0.09
Translation	03010 Ribosome	RER_18640	50S ribosomal protein L5 (rplE)	0.79	1.23	1.73
	03010 Ribosome	RER_18520	50S ribosomal protein L3 (rplC)	1.77	1.48	1.54

03010 Ribosome	RER_18670	30S ribosomal protein S8 (rpsH)	1.61	1.41	1.44
03010 Ribosome	RER_18790	30S ribosomal protein S11 (rpsK)	1.51	0.7	1.31
03010 Ribosome	RER_18700	30S ribosomal protein S5 (rpsE)	0.72	0.54	1.18
03010 Ribosome	RER_18680	50S ribosomal protein L6 (rplF)	-	0.29	1.17
03010 Ribosome	RER_18550	ribosomal protein L2 (rplB)	1.63	1.07	1.17
03010 Ribosome	RER_18800	30S ribosomal protein S4 (rpsD)	0.15	0.35	1.06
03010 Ribosome	RER_58280	50S ribosomal protein L9 (rplI)	0.7	0.85	1
03010 Ribosome	RER_18530	50S ribosomal protein L4 (rplD)	0.39	0.27	0.97
03010 Ribosome	RER_18720	50S ribosomal protein L15 (rplO)	0.24	0.39	0.95
03010 Ribosome	RER_18630	50S ribosomal protein L24 (rplX)	0.36	0.7	0.84
03010 Ribosome	RER_17180	50S ribosomal protein L1 (rplA)	1.3	0.34	0.79
03010 Ribosome	RER_17620	30S ribosomal protein S7 (rpsG)	1.21	1.09	0.78
03010 Ribosome	RER_25420	30S ribosomal protein S2 (rpsB)	0.46	0.16	0.68
03010 Ribosome	RER_24570	50S ribosomal protein L19 (rplS)	0.11	0.28	0.64
03010 Ribosome	RER_19050	30S ribosomal protein S9 (rpsI)	0.82	0.16	0.55
03010 Ribosome	RER_33120	probable 30S ribosomal protein S1 (rpsA)	0.19	0.37	0.53
03010 Ribosome	RER_17170	50S ribosomal protein L11 (rplK)	0.67	0.43	0.5
03010 Ribosome	RER_18620	50S ribosomal protein L14 (rplN)	0.41	0.36	0.48
03010 Ribosome	RER_18590	50S ribosomal protein L16 (rplP)	0.1	0.12	0.47
03010 Ribosome	RER_17200	50S ribosomal protein L10 (rplJ)	1.14	0.12	0.46
03010 Ribosome	RER_18570	50S ribosomal protein L22 (rplV)	0.31	0.06	0.45
03010 Ribosome	RER_26430	30S ribosomal protein S15 (rpsO)	0.24	-	0.43
03010 Ribosome	RER_18580	30S ribosomal protein S3 (rpsC)	0.13	0.3	0.42
03010 Ribosome	RER_19040	50S ribosomal protein L13 (rplM)	0.38	-	0.4
03010 Ribosome	RER_18540	50S ribosomal protein L23 (rplW)	-	0.06	0.36
03010 Ribosome	RER_24440	probable 30S ribosomal protein S16 (rpsP)	0.09	0.07	0.33
03010 Ribosome	RER_17610	30S ribosomal protein S12 (rpsL)	0.14	0.13	0.23
03010 Ribosome	RER_18780	30S ribosomal protein S13 (rpsM)	0.06	0.04	0.2
03010 Ribosome	RER_37930	50S ribosomal protein L27 (rpmA)	0.54	0.11	0.2
03010 Ribosome	RER_18820	50S ribosomal protein L17 (rplQ)	0.27	0.17	0.19
03010 Ribosome	RER_18690	50S ribosomal protein L18 (rplR)	0.11	0.05	0.16

	03010 Ribosome	RER_43250	probable 50S ribosomal protein L25 (rplY)	-	-	0.15
	03010 Ribosome	RER_32940	50S ribosomal protein L35 (rpmI)	0.27	0.05	0.14
	03010 Ribosome	RER_37670	30S ribosomal protein S20 (rpsT)	0.04	-	0.11
	03010 Ribosome	RER_17210	50S ribosomal protein L7/L12 (rplL)	0.07	0.05	0.11
	03010 Ribosome	RER_18610	30S ribosomal protein S17 (rpsQ)	0.04	0.09	0.11
	03010 Ribosome	RER_44060	30S ribosomal protein S18 (rpsR)	0.03	-	0.08
	03010 Ribosome	RER_37940	50S ribosomal protein L21 (rplU)	-	0.16	0.07
	03010 Ribosome	RER_18510	30S ribosomal protein S10 (rpsJ)	0.15	0.09	0.07
	03010 Ribosome	RER_39180	50S ribosomal protein L31 (rpmE)	0.06	0.05	0.06
	03010 Ribosome	RER_18560	30S ribosomal protein S19 (rpsS)	-	0.21	0.05
	03010 Ribosome	RER_58290	probable 30S ribosomal protein S18 (rpsR)	-	-	0.05
	03010 Ribosome	RER_24220	probable 50S ribosomal protein L28 (rpmB)	0.2	-	0.04
	03010 Ribosome	RER_58310	30S ribosomal protein S6 (rpsF)	-	-	0.02
	03010 Ribosome	RER_18650	30S ribosomal protein S14 type Z (rpsZ, rpsN)	-	0.03	-
	00970 Aminoacyl-tRNA biosynthesis	RER_29030	threonyl-tRNA synthetase, ThrRS, threonine--tRNA ligase (thrS)	-	0.05	-
Folding, sorting and degradation	03018 RNA degradation	RER_15260	60 kDa chaperonin, GroEL protein (groEL2)	4.93	6.06	13.53
	03018 RNA degradation	RER_13510	chaperone protein DnaK, Hsp70 (dnaK)	3.07	2.12	2.96
	03018 RNA degradation	RER_19240	60 kDa chaperonin, GroEL protein (groEL1)	0.3	1.02	1.9
	04141 Protein processing in endoplasmic reticulum	RER_50170	chaperone protein HtpG (htpG)	0.36	0.18	1.45
	03018 RNA degradation	RER_42830	enolase, phosphopyruvate hydratase (eno)	0.3	0.08	0.42
	04141 Protein processing in endoplasmic reticulum	RER_12450	putative heat shock protein Hsp18 (hsp18)	-	-	0.37
	03060 Protein export	RER_24420	signal recognition particle protein (ffh)	-	-	0.14
	03018 RNA degradation	RER_26440	guanosine pentaphosphate synthetase/polynucleotide phosphorylase, polyribonucleotide nucleotidyltransferase (gpsI, pnp)	0.05	0.34	0.12
	03018 RNA degradation	RER_40940	probable ATP-dependent RNA helicase DeaD, DEAD-box protein (deaD)	-	-	0.11
	03060 Protein export	RER_29260	putative protein-export membrane protein YajC (yajC)	-	0.04	0.05
	03060 Protein export	RER_29260	putative protein-export membrane protein YajC (yajC)	-	0.04	-
	04122 Sulfur relay system	RER_48100	putative thiosulfate sulfurtransferase, rhodanese	-	0.15	-
	03030 DNA replication	RER_35310	DNA polymerase III alpha subunit (dnaE)	-	-	0.43

Replication and repair	03430 Mismatch repair	RER_35310	DNA polymerase III alpha subunit (dnaE)	-	-	0.43
	03440 Homologous recombination	RER_35310	DNA polymerase III alpha subunit (dnaE)	-	-	0.43
	03030 DNA replication	RER_58300	single-stranded DNA-binding protein (ssb)	0.28	0.09	0.12
	03430 Mismatch repair	RER_58300	single-stranded DNA-binding protein (ssb)	0.28	0.09	0.12
	03440 Homologous recombination	RER_58300	single-stranded DNA-binding protein (ssb)	0.28	0.09	0.12
	03440 Homologous recombination	RER_27530	RecA protein, recombinase A (recA)	-	0.04	0.05
	03440 Homologous recombination	RER_27530	RecA protein, recombinase A (recA)	-	0.04	-
Membrane transport	02010 ABC transporters	RER_27570	glutamate ABC transporter glutamate-binding protein (gluB)	0.69	0.27	0.53
	03070 Bacterial secretion system	RER_24420	signal recognition particle protein (ffh)	-	-	0.14
	02060 Phosphotransferase system (PTS)	RER_39610	phosphotransferase system enzyme IIC component, PTS sugar-specific EIIC component	-	0.13	0.13
	03070 Bacterial secretion system	RER_29260	putative protein-export membrane protein YajC (yajC)	-	0.04	0.05
	03070 Bacterial secretion system	RER_29260	putative protein-export membrane protein YajC (yajC)	-	0.04	-
	02060 Phosphotransferase system (PTS)	RER_39610	phosphotransferase system enzyme IIC component, PTS sugar-specific EIIC component	-	0.13	-
	02060 Phosphotransferase system (PTS)	RER_39630	phosphotransferase system enzyme IIA component, PTS sugar-specific EIIA component	-	0.1	-
	02010 ABC transporters	RER_10320	putative ABC transporter substrate-binding protein	0.06	-	-
Signal transduction	04066 HIF-1 signaling pathway	RER_30290	glyceraldehyde-3-phosphate dehydrogenase (gap)	2.22	1.42	2.25
	04151 PI3K-Akt signaling pathway	RER_50170	chaperone protein HtpG (htpG)	0.36	0.18	1.45
	04066 HIF-1 signaling pathway	RER_42830	enolase, phosphopyruvate hydratase (eno)	0.3	0.08	0.42
	02020 Two-component system	RER_42680	putative beta-ketoadipyl-CoA thiolase	-	0.48	0.2
	02020 Two-component system	RER_36230	glutamine synthetase I, glutamate--ammonia ligase (glnA, glnA1)	-	-	0.11
	04066 HIF-1 signaling pathway	RER_59850	branched-chain alpha-keto acid dehydrogenase E1 beta subunit	-	-	0.08
	02020 Two-component system	RER_12070	acetyl-CoA acetyltransferase, acetoacetyl-CoA thiolase	-	0.04	-
	02020 Two-component system	RER_38940	acetyl-CoA acetyltransferase, acetoacetyl-CoA thiolase	-	0.04	-
	04151 PI3K-Akt signaling pathway	RER_10900	GTP-dependent phosphoenolpyruvate carboxykinase (pckG)	0.03	-	-
Transport and catabolism	04146 Peroxisome	RER_20050	NADP-dependent isocitrate dehydrogenase (icd)	0.13	0.8	0.56
	04146 Peroxisome	RER_14950	probable superoxide dismutase, Cu/Zn-SOD (sodC)	0.81	0.28	0.45
	04146 Peroxisome	RER_40330	probable long-chain fatty-acid--CoA ligase, acyl-CoA synthetase (fadD)	-	0.21	-

Cell growth and death	04112 Cell cycle - Caulobacter	RER_38150	ATP-dependent Clp protease proteolytic subunit ClpP, endopeptidase Clp (clpP)	-	0.16	0.13
	04112 Cell cycle - Caulobacter	RER_35490	cell division protein FtsZ, GTPase (ftsZ)	-	-	0.08
	04112 Cell cycle - Caulobacter	RER_38140	ATP-dependent Clp protease proteolytic subunit ClpP, endopeptidase Clp (clpP)	-	0.04	-
Unclassified		RER_19230	10 kDa chaperonin, GroES protein (groES)	0.81	0.39	1.89
		RER_09370	putative oxidoreductase	1.84	2.07	1.76
		RER_pREL1-02570	putative zinc-containing alcohol dehydrogenase	1.69	0.91	1.46
		RER_04970	putative ABC transporter substrate-binding protein	0.44	-	1.4
		RER_24100	putative DNA-binding protein HU, histone-like protein (hup)	2.87	0.17	1
		RER_25430	elongation factor Ts, EF-Ts (tsf)	0.02	0.23	0.98
		RER_55180	putative AraC family transcriptional regulator	0.4	0.7	0.97
		RER_07700	hypothetical protein	0.08	-	0.89
		RER_02160	mycolyltransferase	13.96	2.3	0.87
		RER_pREL1-02550	conserved hypothetical protein	1.19	1.19	0.84
		RER_04800	CRP/FNR family transcriptional regulator	1.04	0.53	0.61
		RER_pREC1-00490	conserved hypothetical protein	4.6	0.5	0.56
		RER_35390	hypothetical protein	0.15	0.34	0.44
		RER_27870	alkyl hydroperoxide reductase AhpC, alkyl hydroperoxidase C, peroxiredoxin (ahpC)	0.28	0.68	0.43
		RER_05950	putative tellurium resistance protein	0.2	0.06	0.41
		RER_01360	putative 3-hydroxyacyl-CoA dehydrogenase	0.23	0.18	0.39
		RER_41220	2-oxoglutarate dehydrogenase E1 component (sucA, odhA)	-	0.05	0.37
		RER_41730	aromatic amino acid transport protein (aroP)	0.25	1.59	0.37
		RER_17630	elongation factor G, EF-G (fusA)	0.06	0.35	0.35
		RER_pREL1-02600	cytochrome P450	0.31	0.36	0.35
		RER_41180	conserved hypothetical protein	-	-	0.35
		RER_pREL1-01540	phosphate ABC transporter phosphate-binding protein (pstS)	0.45	-	0.34
		RER_pREL1-02830	cytochrome P450	0.06	1.03	0.29
		RER_06850	hypothetical protein	0.49	0.38	0.27
		RER_11630	hypothetical protein	0.04	-	0.24
		RER_00210	putative CdaR family transcriptional regulator	-	-	0.23

RER_42290	hypothetical protein	0.11	-	0.2
RER_36350	mycolyltransferase	3.3	0.91	0.2
RER_54170	probable mycolyltransferase	2.96	0.74	0.2
RER_30750	enoyl-[acyl-carrier-protein] reductase (fabI)	-	-	0.19
RER_23310	conserved hypothetical protein	0.08	0.2	0.19
RER_26320	translation initiation factor IF-2 (infB)	-	0.03	0.17
RER_47220	putative ATP-dependent Clp protease ATP-binding subunit ClpC (clpC)	-	-	0.16
RER_46820	hypothetical protein	-	-	0.16
RER_35440	conserved hypothetical protein	-	-	0.14
RER_36210	conserved hypothetical protein	0.21	-	0.13
RER_52420	putative acyl-CoA dehydrogenase	-	-	0.13
RER_47410	putative organic hydroperoxide resistance protein (ohr)	-	-	0.12
RER_01740	putative ferritin (ftn)	-	-	0.12
RER_44690	conserved hypothetical protein	0.19	0.09	0.12
RER_pREL1-02560	fatty-acid--CoA ligase, acyl-CoA synthetase (fadD)	0.69	-	0.12
RER_34140	putative membrane protein	-	-	0.12
RER_00120	conserved hypothetical protein	0.4	-	0.11
RER_06460	putative ATP-dependent Clp protease ATP-binding subunit ClpC (clpC)	0.31	0.05	0.1
RER_38330	putative ABC transporter ATP-binding protein	-	-	0.1
RER_41420	putative two-component histidine kinase	-	0.35	0.1
RER_41620	putative methyltransferase	-	0.13	0.1
RER_pREC1-00400	conserved hypothetical protein	-	-	0.09
RER_31950	hypothetical protein	-	-	0.09
RER_29220	conserved hypothetical protein	0.39	0.11	0.08
RER_03650	putative LysR family transcriptional regulator	-	-	0.08
RER_29970	elongation factor P, EF-P (efp)	0.08	-	0.07
RER_38160	trigger factor (tig)	-	0.06	0.07
RER_18370	putative non-ribosomal peptide synthetase	-	-	0.07
RER_pREL1-02750	putative acyltransferase	-	-	0.07

RER_18890	probable S8 family peptidase	-	-	0.07
RER_21790	acyl-CoA desaturase, steroyl-CoA delta(9)-desaturase	-	-	0.07
RER_41920	probable GTP-binding protein TypA (typA, bipA)	-	-	0.07
RER_42610	transcription elongation factor GreA (greA)	-	-	0.06
RER_41130	putative drug resistance efflux protein	-	-	0.06
RER_49020	putative TetR family transcriptional regulator	-	-	0.06
RER_15890	putative heparin-binding hemagglutinin	-	0.16	0.05
RER_12710	probable cold shock protein	0.11	-	0.05
RER_00330	conserved hypothetical protein	-	-	0.05
RER_05110	probable cold shock protein	0.03	-	0.05
RER_35260	putative TetR family transcriptional regulator	-	-	0.05
RER_38530	putative TetR family transcriptional regulator	-	-	0.05
RER_13720	conserved hypothetical protein	-	-	0.04
RER_40770	putative acyl-CoA carboxylase	-	-	0.04
RER_31380	conserved hypothetical protein	0.05	1.1	0.04
RER_pREC1-00050	hypothetical protein	-	-	0.04
RER_28540	probable peptide methionine sulfoxide reductase MsrB (msrB)	-	-	0.03
RER_pREL1-01680	conserved hypothetical protein	-	-	0.03
RER_37780	conserved hypothetical protein	-	-	0.02
RER_pREC1-00500	conserved hypothetical protein	2.08	-	0.01
RER_02750	putative membrane protein	0.92	0.06	-
RER_04680	conserved hypothetical protein	0.06	0.09	-
RER_13390	putative S1 family peptidase	0.07	0.05	-
RER_27420	conserved hypothetical protein	0.21	0.13	-
RER_36730	acyl carrier protein (acpM, acpP)	0.03	0.05	-
RER_37100	putative Fur family transcriptional regulator	0.5	0.15	-
RER_42380	conserved hypothetical protein	0.16	0.04	-
RER_48260	conserved hypothetical protein	2.39	0.24	-
RER_00100	DNA gyrase subunit A (gyrA)	-	0.09	-
RER_00170	peptidyl-prolyl cis-trans isomerase	-	0.19	-
RER_00570	hypothetical protein	-	0.06	-

RER_02660	putative membrane protein	-	0.23	-
RER_02720	hypothetical protein	-	0.05	-
RER_02790	hypothetical protein	-	0.03	-
RER_04530	putative oxidoreductase	-	2.95	-
RER_06040	putative tellurium resistance protein	-	0.09	-
RER_06640	conserved hypothetical protein	-	0.04	-
RER_07490	rubredoxin reductase (rubB)	-	1.02	-
RER_08230	putative ABC transporter substrate-binding protein	-	0.14	-
RER_09220	conserved hypothetical protein	-	0.11	-
RER_10650	conserved hypothetical protein	-	0.52	-
RER_12570	hypothetical protein	-	1.07	-
RER_15250	hypothetical protein	-	0.32	-
RER_15910	putative hydrolase	-	0.04	-
RER_16250	hypothetical membrane protein	-	1.15	-
RER_16590	putative ABC transporter substrate-binding protein	-	0.4	-
RER_16920	hypothetical protein	-	1.82	-
RER_17600	conserved hypothetical protein	-	1.12	-
RER_19720	putative oxidoreductase	-	0.82	-
RER_20960	conserved hypothetical protein	-	0.96	-
RER_21720	conserved hypothetical protein	-	0.05	-
RER_21780	NADPH oxidoreductase	-	0.03	-
RER_23390	molybdenum cofactor biosynthesis bifunctional protein MoaCB (moaCB)	-	0.01	-
RER_23650	putative methyltransferase	-	0.33	-
RER_27050	putative MFS transporter	-	0.19	-
RER_27300	hypothetical protein	-	0.03	-
RER_27680	conserved hypothetical membrane protein	-	0.66	-
RER_30420	putative OxPP cycle protein OpcA (opcA)	-	2	-
RER_30570	putative iron-sulfur cluster assembly protein SufC, ABC-type ATPase (sufC)	-	0.04	-
RER_32120	conserved hypothetical protein	-	0.04	-

RER_33060	conserved hypothetical protein	-	0.08	-
RER_36740	3-oxoacyl-[acyl-carrier-protein] synthase II, beta-ketoacyl-ACP synthase II (fabF, kasA)	-	0.08	-
RER_37400	chaperone protein DnaJ (dnaJ)	-	0.23	-
RER_38160	trigger factor (tig)	-	0.06	-
RER_38240	conserved hypothetical protein	-	0.03	-
RER_38250	conserved hypothetical protein	-	0.15	-
RER_41220	2-oxoglutarate dehydrogenase E1 component (sucA, odhA)	-	0.05	-
RER_41410	conserved hypothetical protein	-	0.05	-
RER_42070	conserved hypothetical protein	-	0.36	-
RER_44660	probable NarL family two-component response regulator	-	0.08	-
RER_45980	hypothetical protein	-	0.07	-
RER_46110	FKBP-type peptidyl-prolyl cis-trans isomerase	-	0.02	-
RER_46390	probable resuscitation-promoting factor (rpf)	-	0.06	-
RER_49470	putative ABC transporter ATP-binding protein	-	0.07	-
RER_50370	putative ABC transporter permease protein	-	0.13	-
RER_52580	acyl-CoA dehydrogenase (fadE)	-	0.14	-
RER_54770	putative ArsR family transcriptional regulator	-	0.48	-
RER_55960	putative oxidoreductase	-	0.01	-
RER_56230	putative 3-hydroxyacyl-CoA dehydrogenase	-	0.05	-
RER_56880	putative hydrolase	-	1.87	-
RER_57630	putative MerR family transcriptional regulator	-	0.25	-
RER_59430	putative acid--CoA ligase	-	0.08	-
RER_59700	hypothetical protein	-	0.13	-
RER_pREC1-00280	conserved hypothetical protein	-	0.16	-
RER_pREC1-00750	transposase (tnpA)	-	0.12	-
RER_pREL1-00940	conserved hypothetical protein		0.45	-
RER_00620	putative CPA1 family transporter	0.53	-	-
RER_01310	conserved hypothetical protein	0.43	-	-
RER_01800	conserved hypothetical membrane protein	0.12	-	-
RER_02170	mycolyltransferase (csp1)	1.4	-	-

RER_02210	probable polyketide synthase Pks13, type I polyketide synthase (pks13)	0.03	-	-
RER_08480	putative iron-containing alcohol dehydrogenase	0.08	-	-
RER_11110	conserved hypothetical protein	0.14	-	-
RER_13910	chaperone ClpB (clpB)	0.17	-	-
RER_15370	putative mycolyltransferase	0.26	-	-
RER_15970	hypothetical protein	0.09	-	-
RER_17110	conserved hypothetical protein	0.32	-	-
RER_18410	putative mycolyltransferase	1.62	-	-
RER_18760	translation initiation factor IF-1 (infA)	0.03	-	-
RER_24560	conserved hypothetical membrane protein	0.75	-	-
RER_26470	hypothetical protein	0.57	-	-
RER_28200	conserved hypothetical protein	0.07	-	-
RER_29950	hypothetical membrane protein	0.09	-	-
RER_32010	conserved hypothetical protein	0.18	-	-
RER_33030	hypothetical protein	0.1	-	-
RER_35890	conserved hypothetical protein	0.02	-	-
RER_37800	putative oxidoreductase	0.05	-	-
RER_38660	conserved hypothetical protein	0.09	-	-
RER_40130	putative mannose-binding protein, lectin	0.1	-	-
RER_41960	conserved hypothetical protein	0.24	-	-
RER_44310	conserved hypothetical protein	0.05	-	-
RER_44680	putative F420-dependent oxidoreductase	0.12	-	-
RER_44710	conserved hypothetical protein	0.06	-	-
RER_45180	conserved hypothetical protein	0.04	-	-
RER_48960	putative MFS transporter	0.18	-	-
RER_55800	putative fatty-acid--CoA ligase, acyl-CoA synthetase	0.09	-	-
RER_59030	putative ABC transporter substrate-binding protein	0.06	-	-
RER_pREL1-01860	putative lipase	0.31	-	-
RER_pREL1-02580	aldehyde dehydrogenase	0.02	-	-
RER_pREL1-02590	2Fe-2S ferredoxin	0.15	-	-

Shotgun proteomic analysis results of the samples resolved in the gel shown in Fig. S1A. The data indicate the relative percent of total protein detected by LC-MS/MS. Proteins under all conditions tested are categorized based on the KO IDs, and listed with reference in decreasing order of relative abundance of C19. A bar (-) means not detected.

Supplemental figure legends

Fig. S1. Protein analysis. (A) SDS-PAGE analysis. “None” indicates total proteins extracted from *R. erythropolis* PR4 cells grown in IB medium, “C12” indicates total proteins extracted from PR4 cells grown in IB medium containing *n*-dodecane (C12), and “C19” indicates total proteins extracted from PR4 cells grown in IB medium containing pristane (C19). (B) GroEL2 expression by western blotting analysis of GroEL2 using anti-GroEL2 polyclonal antibody. Numbers indicate relative amounts of GroEL2 compared to the control condition (None), which was taken as 1.

Fig. S2. Localization of the PR4 (pK4-ΔEL2-1) strain in two phase cultures containing alkanes of various carbon chain length (upper series). Lower series shows phase-contrast micrographs of hydrocarbon droplets illustrating differences in bacterial localization. “AL” and “AQ” indicate the alkane phase and the aqueous supernatant phase, respectively. All photographs show the same magnification. Scale bar = 10 μm.

Fig. S1.

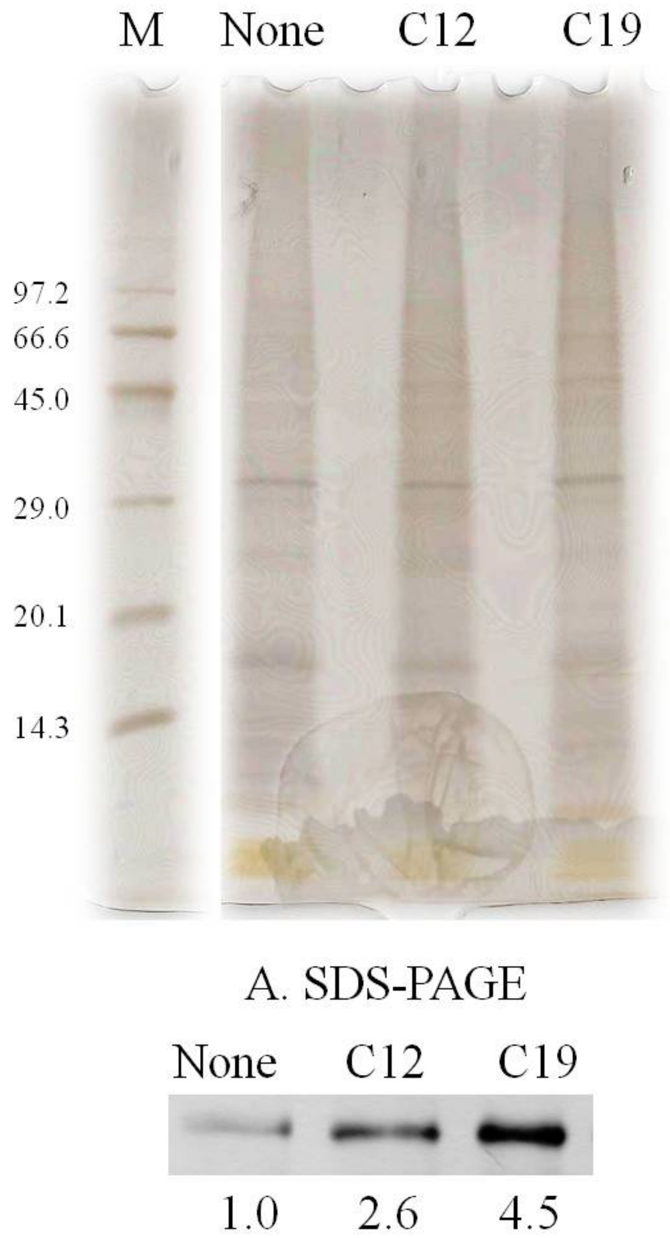

A. SDS-PAGE

B. Expression of GroEL2 by western blotting

Fig. S2.

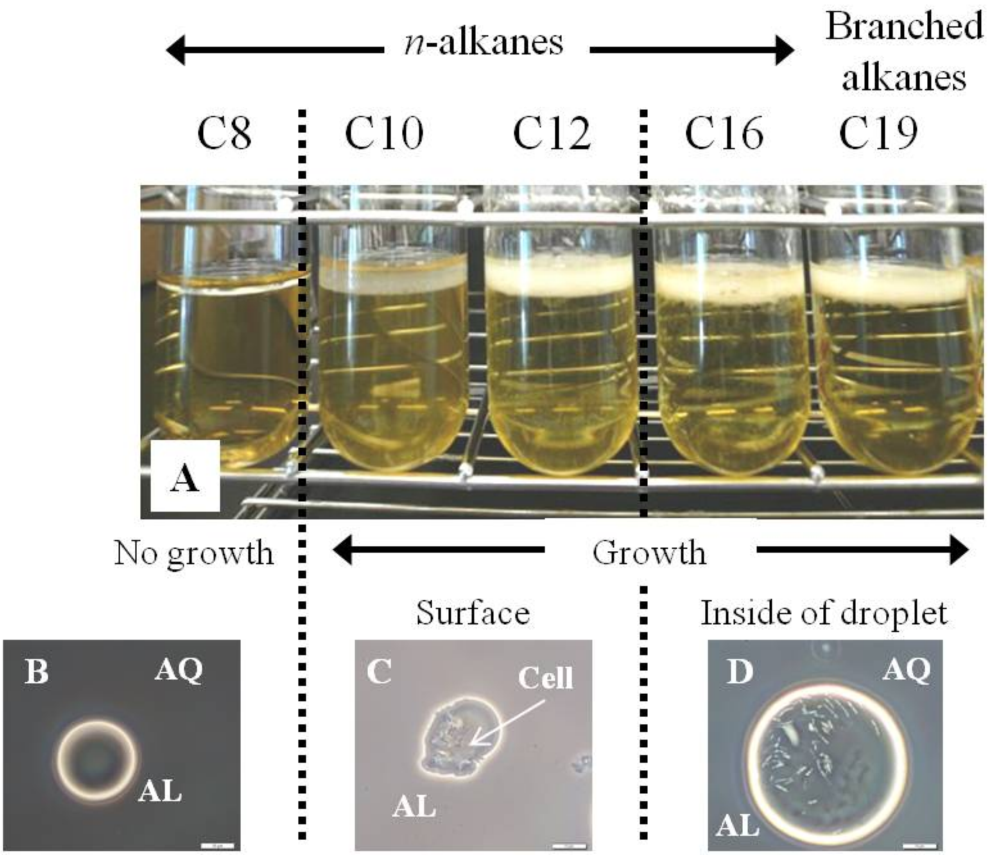

n-alkanes
Branched alkanes
C8
C10
C12
C16
C19
A
No growth
Growth
Surface
Inside of droplet
B
AQ
AL
C
Cell
AL
D
AQ
AL
